# Theoretical Analysis of Interferometer Wave Front Tilt and Fringe Radiant Flux on a Rectangular Photodetector

**DOI:** 10.3390/s130911861

**Published:** 2013-09-06

**Authors:** Robert Smith, Franz Konstantin Fuss

**Affiliations:** School of Aerospace, Mechanical and Manufacturing Engineering, RMIT University, Plenty Road, Melbourne 3083, Australia

**Keywords:** photodetector, fringe analysis, radiant flux, interferometry

## Abstract

This paper is a theoretical analysis of mirror tilt in a Michelson interferometer and its effect on the radiant flux over the active area of a rectangular photodetector or image sensor pixel. It is relevant to sensor applications using homodyne interferometry where these opto-electronic devices are employed for partial fringe counting. Formulas are derived for radiant flux across the detector for variable location within the fringe pattern and with varying wave front angle. The results indicate that the flux is a damped sine function of the wave front angle, with a decay constant of the ratio of wavelength to detector width. The modulation amplitude of the dynamic fringe pattern reduces to zero at wave front angles that are an integer multiple of this ratio and the results show that the polarity of the radiant flux changes exclusively at these multiples. Varying tilt angle causes radiant flux oscillations under an envelope curve, the frequency of which is dependent on the location of the detector with the fringe pattern. It is also shown that a fringe count of zero can be obtained for specific photodetector locations and wave front angles where the combined effect of fringe contraction and fringe tilt can have equal and opposite effects. Fringe tilt as a result of a wave front angle of 0.05° can introduce a phase measurement difference of 16° between a photodetector/pixel located 20 mm and one located 100 mm from the optical origin.

## 1. Introduction

**Figure 1.**
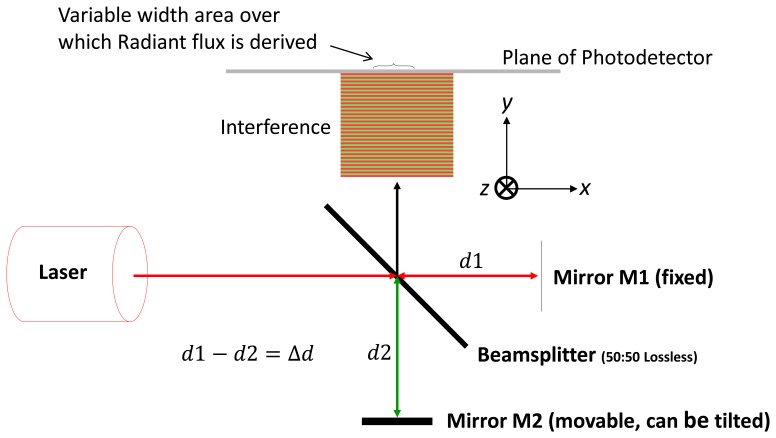
Michelson interferometer.

The Michelson interferometer [1] shown in Figure 1 has been used extensively in the field of metrology, most famously in the Michelson-Morley experiment [2]. The measurement is based on detecting and measuring the number of complete and partial fringes resulting from translation of one of the reflecting mirrors and relating the result to the light-source wavelength.

Discrete photodetectors and image sensors are commonly used to detect the sinusoidal pulsing fringe pattern. The radiant flux of the fringe pattern incident on the device active area is mathematically derived by integrating the irradiance over the circular aperture of the light source [3]; over a circular aperture of the interferogram [4–10]; over a square/rectangular aperture of the interferogram [8,11]. Effectively, the active area of the photodetector performs the same function on the irradiance giving an output proportional to the radiant flux.

When using a well collimated beam and plane flat mirrors that are not perfectly aligned, *i.e.*, tilted, fringe lines of equal inclination, width and spacing are produced that contract as the tilt angle is increased and expand as the tilt angle is reduced, having a significant effect on the radiant flux over the active area. This change in radiant flux can be a source of measurement error [3–9] when unconsidered in applications [12–16].

As wave front angle increases, the modulation amplitude of the dynamic fringing reduces and at a specific tilt angle the modulation amplitude of the radiant flux becomes zero [3,5–11,17]. The modulation amplitude is found to decay as a cardinal sine function with a rate of decay proportional to the area of the photodetector and inversely proportional to wavelength.

To overcome or minimise mirror tilt prevalent with flat plane mirrors, corner cube retro-reflectors [8,9], cat-eye reflectors [18] or alternate interferometers [11] are used.

The behaviour of the radiant flux with varying wave front angle is also affected with varying photodetector distance from the central axis of the interferometer and varying its distance from the optical model origin. Analysis of the radiant flux with varying wave front in conjunction with photodetector area, distance from beam centre, distance from origin and wavelength appears not to be covered in the literature although [17] has included distance of the photodetector from the optical model origin specifically to determine the maximum offset angle for a beam-tilting spatial modulation interferometer.

Despite modulation amplitude *vs.* wave front angle being well understood, to the best of our knowledge, the following analysis is not covered in the literature, *i.e.*, the behaviour of the radiant flux: for a rectangular aperture for varying wave front angle beyond the first modulation zero; over varying active area widths for varying distances from beam centre, e.g., row of pixels across an image sensor; for varying active widths and distances from the tilted mirror; with fringe contraction speed and mirror tilt; with decay constant.

This paper addresses these issues, specifically:

- Behaviour of the radiant flux for variable wave front angle as a function of photodetector width and position within the fringe pattern;
- Behaviour of the radiant flux on two identical photodetectors adjacent each other;
- Magnitude of the radiant flux at wave front angle(s) of equal radiant flux;
- Polarity reversal of the radiant flux beyond specific wave front angles;
- Behaviour of the radiant flux for variable wave front angle with variable distance of the photodetector from the tilting mirror;
- Speed of transition of the fringe lines across the photodetector for variable wave front angle;
- Speed of fringe line tilt across the photodetector for variable wave front angle;
- Damping function constant of the radiant flux for variable wave front angle.

The relevance of this theoretical analysis is to make evident how these other factors may have an undesirable effect on sensor applications using homodyne interferometry where photodetectors or image sensors are employed to sense small fractions of a fringe to achieve extremely high resolutions of measurement. It goes beyond the adverse effect of modulation amplitude reduction due to increasing wave front angle [3,5–11,17] and introduces what have been termed *primary nodes*. The results from the mathematical analysis describes how the radiant flux behaves when the five parameters; wave front angle, wave length, photodetector width and position (*x*, *y*) are varied independently and concurrently, and how this behaviour can introduce fringe counting errors.

## 2. Mathematical Analysis

The analysis is carried out based on a conventional Michelson Interferometer that is configured as shown in Figure 1 with the following configuration constraints:

- Light source is a collimated monochromatic beam;
- Wave fronts over the area of the photodetector are approximated to be plane waves;
- Flat plane mirrors are used to reflect the transmitted and reflected beams back to the beamsplitter;
- Beamsplitter is lossless and is non-polarising and creates a transmitted and reflected beam of equal amplitude.

**Figure 2.**
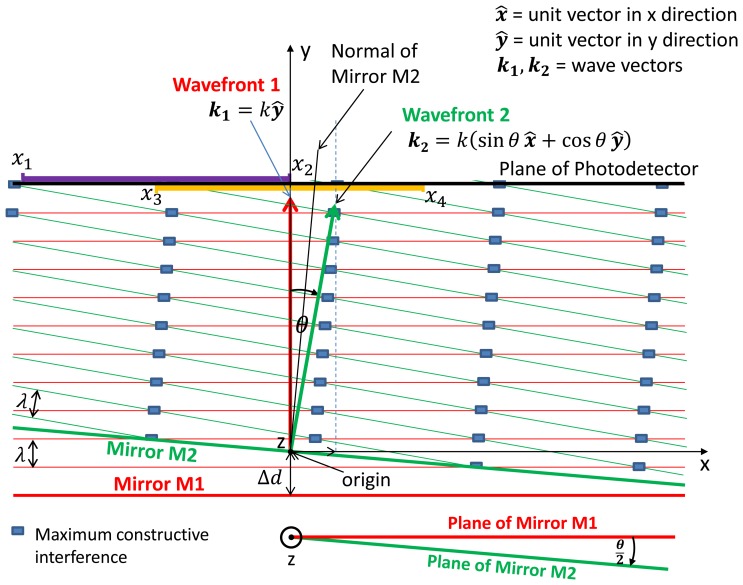
Wave fronts 1 and 2 with Mirror M2 tilted at angle *θ*/2 about the *z*-axis.

The two wave fronts are orientated as depicted in Figure 2 and all calculations are based on the following constraints:

- *y* axis is taken to be normal to wave front 1;
- Origin of the Cartesian coordinate system is the point at which the centre of the incident beam is reflected by mirror M2;
- Mirror M2 tilts only about the *z*-axis;
- Mirror M2 translates only along the *y*-axis;
- Plane of the photodetector remains orthogonal to the *y* axis;
- Shape of the active area of the photodetector is rectangular with variable side length *s* in the *x* direction and fixed side length *z* (set to unity) in the *z* direction;
- Fringe pattern irradiates the entire active area of the photodetector;
- Output of the photodetector is assumed to be a 1:1 linear function of the incident radiant flux;
- Distance to the photodetector from mirror M2 is variable.

The mathematical analysis is divided into the following subsections, the outcome of which is studied further in the Results section:

1. Derivation of the equation for radiant flux from irradiance of the fringe pattern;
2. Identification of specific wave front angles *θ_n_* with invariable radiant flux for two displaced photodetectors of equal size active areas;
3. Determination of the magnitude of the radiant flux at specific wave front angles *θ_n_*;
4. Determination of the linear equation defining the profile of the fringe pattern in the *x-y* plane;
5. Determination of the speed of the fringe lines with variable wave front angle *θ*;
6. Determination of the damping function of the radiant flux with variable wave front angle *θ*.

Note: The angle *θ* in this paper refers to the angle that Wave Front 2 makes with Wave Front 1. The tilt angle of Mirror M2 is therefore *θ*/2 relative to Mirror M1.

### 2.1. Derivation of the Equation for Radiant Flux

The electric field of a plane wave is given by Equation (1) [19]:

(1) $$E\left(r,t\right)=E_{0}e^{i(k.r-\omega t)}$$

where **E** is the time (*t*) dependent electric field, **r** is the unit vector of the electric field in 3 dimensional space, *i.e.*, **r** =*x***x̂** + *y***ŷ** + *z***ẑ** and **x̂**, **ŷ** and **ẑ** are unit vectors along the *x*, *y* and *z* axes, **E_0_** is the vector amplitude of the wave, **k** is the wave vector where **k** = *k***u,** where **u** is the unit vector defining the direction of propagation [20] and |**k**| = 2*π*/ *λ, λ*, is the wavelength of the light source, *k* is the wave number and *ω* is the angular frequency of the wave.

Figure 2 depicts the linear optical equivalent of the Michelson interferometer with the virtual source wave front approaching mirrors M1 and M2 from the top of the figure. With reference to the origin, the reflected wave fronts 1 and 2 from respective mirrors have wave vectors **k_1_** and **k_2_**:

(2) $${\text{k}}_{1}=k\hat{\text{y}}$$

(3) $${\text{k}}_{2}=k\sin\theta \hat{\text{x}}+k\cos\theta \hat{\text{y}}$$

Also depicted in Figure 2, the source wave front travels a distance Δ*d* further to M1 creating an optical path difference (OPD) between the wave fronts and a phase lag of *k*2Δ*d* relative to wave front 2.

The sum of the electric fields of wave fronts 1 and 2 is therefore:

(4) $$E_{\text{sum}}\left(r,t\right)=E_{0}e^{i\left(k_{1}\cdot r-k2\Delta d-\omega t\right)}+E_{0}e^{i\left(k_{2}\cdot r-\omega t\right)}$$

(5) $$E_{\text{sum}}\left(x,y,z\right)=E_{0}e^{-i\omega t}\left(e^{ik\left(y-2\Delta d\right)}+e^{i\left(k\left(y\cos\theta +x\sin\theta \right)\right)}\right)$$

The irradiance *I* of an electric field is given by Equation (6) and is the radiant flux of the electric field delivered per area to a given surface with units Wm^−2^, *i.e.*, radiant flux density:

(6) $$I=\left(\frac{n_{RI}\epsilon_{0}c}{2}\right)E_{\text{sum}}\cdot E_{\text{sum}}^{*}$$

where *n_RI_* is the refractive index of the medium, *c* is the speed of light in vacuum, *ε*_0_ is the vacuum permittivity, and $E_{\text{sum}}^{*}$ the complex conjugate of *E_sum_*:

(7) $$I=2\left(\frac{n_{RI}\epsilon_{0}c}{2}\right){E_{0}}^{2}\left(1+\cos\left(k\left(y-2\Delta d-y\cos\theta -x\sin\theta \right)\right)\right)$$

Equation (7) indicates that the irradiance at a point (*x, y*) in the fringe pattern created by wave fronts 1 and 2 is dependent on the values of *x* and *y*, wave number *k*, which is a function of wavelength, the angle *θ* between the wave fronts and the optical path difference 2Δ*d*.

If Equation (7) is integrated along the *x*-axis between arbitrary points *x*_1_ and *x*_2_ and then multiplied by side length *z* in the *z*-direction to create an area across the photodetector, the solution is the radiant flux incident on a rectangle of side lengths *x*_2_ − *x*_1_ = *s* and *z*. As mirror M2 is only tilted about the *z*-axis, variable *z* does not need to be included in the integration as it behaves purely as a multiplier to the solution of the integration along the *x*-axis. Therefore:

(8) $$\begin{array}{c} \Phi_{e}=z\cdot \int_{x}\text{I dx}=2\left(\frac{n_{RI}\epsilon_{0}c}{2}\right){E_{0}}^{2}z\int_{x_{1}}^{x_{2}}\left(1+\cos\left(k\left(y-2\Delta d-y\cos\theta -x\sin\theta \right)\right)\right)dx\\ =2\left(\frac{n_{RI}\epsilon_{0}c}{2}\right){E_{0}}^{2}z\cdot {\left|\frac{1}{k\sin\theta }\left(\sin\left(k\left(x\sin\theta +y\cos\theta -y+2\Delta d\right)\right)+kx\sin\theta \right)\right|}_{x_{1}}^{x_{2}} \end{array}$$

The radiant flux Φ_e_ given by Equation (8) is expressed in Watts (W) and is the total radiant power of the interference beam incident on the defined rectangular active area of the photodetector. At *θ* = 0, Φ_e_ = 0/0 which is indeterminate, therefore applying L'Hôpital's rule to the integral solution of Equation (8) for *θ* → 0 returns:

(9) $$\underset{\theta \to 0}{\text{lim}}\frac{\left(\sin\left(k\left(x\sin\theta +y\cos\theta -y+2\Delta d\right)\right)+kx\sin\theta \right)}{k\sin\theta }=x\left(1+\cos\left(k2\Delta d\right)\right)$$

Therefore, as *θ* → 0, the radiant flux derived in Equation (8) tends to:

(10) $$\Phi_{e\left(\theta \to 0\right)}=2\left(\frac{n_{RI}\epsilon_{0}c}{2}\right){E_{0}}^{2}z\cdot s\left(1+\cos\left(k2\Delta d\right)\right)$$

The radiant flux in Equation (10) is a maximum when cos(*k*2Δ*d*) = 1, *i.e.*, when 2Δ*d* = *n_f_λ*, where *n_f_* is an integer equivalent to the number of fringe lines and 2Δ*d* is the optical path difference. Whenever the OPD is an integer multiple of the wavelength, the two wave fronts in Figure 2 are in phase with one another resulting in maximum radiant flux, *i.e.*:

(11) $$\Phi_{e\left(\theta \to 0,2\Delta d=n_{f}\lambda \right)}=2\left(\frac{n_{RI}\epsilon_{0}c}{2}\right){E_{0}}^{2}z\cdot 2s$$

**Figure 3.**
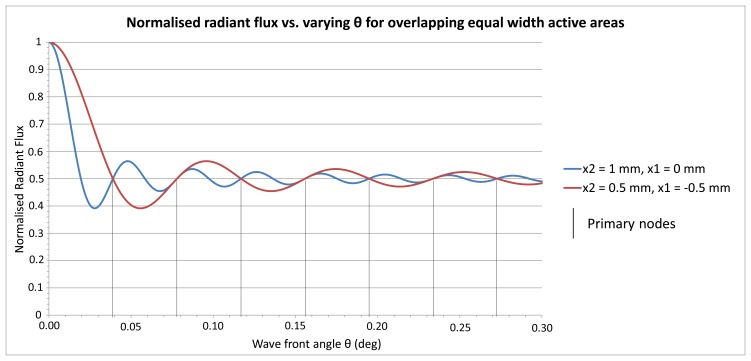
Normalised radiant flux *vs.* varying *θ* with *y* = 0, *λ* = 680 nm, *Δd* = 0, integral width *s* = 1 mm, for integral boundaries *x*_2_ = 0.5 mm & *x*_1_ = −0.5 mm and *x*_2_ = 1 mm & *x*_1_ = 0 mm.

To demonstrate the behaviour of the radiant flux over differing integral boundaries, Figure 3 shows the radiant flux curves for two sets of integral boundaries that are equal in length with assigned variables defined as follows that have been substituted in Equation (10); *y* = 0 m, *λ* = 680 × 10^−9^ m therefore *k* = 9,239,978, Δ*d* = 0 m, integral width *s* = 0.001 m, red curve integral boundaries *x*_2_ = 0.0005 m, *x*_1_ = −0.0005 m, blue curve integral boundaries *x*_2_ = 0.001 m, *x*_1_ = 0 m.

It can be seen from the Figure 3 that there are node points at half the normalised radiant flux that are cyclic, which have been termed *primary nodes*, and this phenomenon is explored further below. What is also noticeable is the two curves converge as *θ* → 0 as predicted in Equation (10).

### 2.2. Identifying Specific Wave Front Angles θ_n_ with Invariable Φ_e_ for Two Sets of Integral Boundaries

To analyse the effect of mirror tilt angle on two separate rectangular areas of equal size and determine the node points observed in Figure 3, consider only the integral solution of Equation (8). If we define the two intervals along the *x*-axis with upper and lower limits *x*_1_, *x*_2_ & *x*_3_, *x*_4_ such that *x*_2_ – *x*_1_ = *x*_4_ – *x*_3_ = *s* and substitute in the Equation (8) we get after simplification Equations (12) and (13):

(12) $$\Phi_{e\left(x_{1},x_{2}\right)}\propto \frac{1}{k\sin\theta }\left[\sin\left(k\left(x_{2}\sin\theta +y\cos\theta -y+2\Delta d\right)\right)+kx_{2}\sin\theta -\sin\left(k\left(x_{1}\sin\theta +y\cos\theta -y+2\Delta d\right)\right)-kx_{1}\sin\theta \right]$$

(13) $$\Phi_{e\left(x_{3},x_{4}\right)}\propto \frac{1}{k\sin\theta }\left[\sin\left(k\left(x_{4}\sin\theta +y\cos\theta -y+2\Delta d\right)\right)+kx_{4}\sin\theta -\sin\left(k\left(x_{3}\sin\theta +y\cos\theta -y+2\Delta d\right)\right)-kx_{3}\sin\theta \right]$$

To solve for *θ* and *x*, let Equation (12) = Equation (13), applying trigonometrical identities and simplifying yields:

(14) $$\sin\left(\frac{ks\sin\theta }{2}\right)\cdot \left[\cos\left(\frac{k\left(\left(x_{2}+x_{1}\right)\sin\theta +2y\cos\theta -2y+4\Delta d\right)}{2}\right)-\cos\left(\frac{k\left(\left(x_{4}+x_{3}\right)\sin\theta +2y\cos\theta -2y+4\Delta d\right)}{2}\right)\right]=0$$

To obtain the node points that satisfy Equation (14) for the two intervals defined above, *i.e.*, *x*_2_ – *x*_1_ = *x*_4_ – *x*_3_ = *s*, values need to be assigned to these boundary limits. For example, let *x*_1_, *x*_2_ & *x*_3_, *x*_4_ be the two intervals depicted along the plane of the photodetector in Figure 2 with values defined as *x*_1_ = −*s; x*_2_ = 0; *x*_3_ = −*s*/*2; x*_4_ = *s*/*2*.

Substituting these values in Equation (14) and simplifying yields:

(15) $$\sin\left(\frac{ks\sin\theta }{2}\right)\cdot \left[\cos\left(\frac{k\left(\left(-s\right)\sin\theta +2y\cos\theta -2y+4\Delta d\right)}{2}\right)-\cos\left(\frac{k\left(2y\cos\theta -2y+4\Delta d\right)}{2}\right)\right]=0$$

The equality of Equation (15) is satisfied if:

(a) $\sin\left(\frac{ks\sin\theta }{2}\right)=0$ and/ or
(b) $\cos\left(\frac{k\left(\left(-s\right)\sin\theta +2y\cos\theta -2y+4\Delta d\right)}{2}\right)-\cos\left(\frac{k\left(2y\cos\theta -2y+4\Delta d\right)}{2}\right)=0$

Solving:

(a) is satisfied when (*ks* sin *θ*)/2 = *n_p_π*, where *n_p_* is an integer and is the number of what is termed a *primary node* (see Figure 3), resulting in:
  (16) $$\sin\theta_{n_{p}}=\frac{2n_{p}\pi }{k_{s}}\text{or}\theta_{n_{p}}=\frac{n_{p}\lambda }{s}$$
  If small angles are considered, implying *sin θ_np_* = *θ_np_*, where the intersection of the two radiant flux curves at *θ_np_* is called a *primary node* and *θ_np_* is called the *primary node* angle
(b) is satisfied when
  (17) $$\cos\left(\frac{k\left(\left(-s\right)\sin\theta +2y\cos\theta -2y+4\Delta d\right)}{2}\right)=\cos\left(\frac{k\left(2y\cos\theta -2y+4\Delta d\right)}{2}\right)$$

There are two solutions that satisfy Equation (17):

- (−*s*) sin *θ* = 0 therefore sin *θ*_0_ = 0 where *θ*_0_ = 0. As only small angles are of concern, *θ* = *nπ* is irrelevant. Note that *θ*_0_ = 0 is co-incident with *primary node n_p_* = 0 from Equation (16).
- The cosines are identical for *n_s_π* ± *Δ*/2, where *Δ* is a phase shift and *n_s_* is an integer related to *secondary nodes*, *i.e.*,:
  (18) $$\cos\left(n_{s}\pi +\frac{\delta }{2}\right)=\cos\left(n_{s}\pi -\frac{\delta }{2}\right)$$

*n_s_* can be zero if the data is mirrored about *θ* = 0 and *n_s_* = 1 if the data is mirrored about *θ* = *π*.

The occurrence of secondary nodes is unique and specific to the defined integral boundaries *x*_1_, *x*_2_ & *x*_3_, *x*_4_, the values of *k*, *y* and Δ*d*. Consequently, the cosine expression in Equation (14) has to be solved accordingly with its own set of boundary conditions. The wave front angle at secondary node angles *θ_ns_* is incidental, unlike at *primary nodes*, which is cyclic and dependent only on *k* and *s* (sine expression in Equation (14)).

### 2.3. Determination of the Magnitude of Φ_e_ at Wave Front Angles θ_np_

To determine the magnitude of the radiant flux of the fringe pattern at the *primary nodes* we solve Equations (12) and (13) independently for the intervals previously defined *i.e.*, *x*_1_ = −*s; x*_2_ = 0; *x*_3_ = −*s*/2; *x*_4_ = *s*/2. Beginning with Equation (12):

(19) $$\Phi_{e\left(x_{1},x_{2}\right)}\propto \frac{\sin\left(k\left(y\cos\theta -y+2\Delta d\right)\right)-\sin\left(k\left(-s\sin\theta +y\cos\theta -y+2\Delta d\right)\right)+ks\sin\theta }{k\sin\theta }$$

For small angles sin *θ* = *θ*, therefore Equation (19) becomes:

(20) $$\Phi_{e\left(x_{1},x_{2}\right)}\propto \frac{\sin\left(k\left(y\cos\theta -y+2\Delta d\right)\right)-\sin\left(k\left(-s\theta +y\cos\theta -y+2\Delta d\right)\right)+ks\theta }{k\theta }$$

At *θ*_0_, Equation (20) reduces to 0/0, which is indeterminate. Therefore applying L'Hôpital's rule:

(21) $$\underset{\theta \to 0}{\lim}\frac{\sin\left(k\left(y\cos\theta -y+2\Delta d\right)\right)-\sin\left(k\left(-s\theta +y\cos\theta -y+2\Delta d\right)\right)+ks\theta }{k\theta }=s\left(\cos\left(k2\Delta d\right)+1\right)$$

Repeating the above for Equation (13) yields the same result.

Equation (21) shows that as *θ* → 0 the radiant flux becomes equal for the integral boundaries *x*_1_ = −*s; x*_2_ = 0; *x*_3_ = −*s*/2; *x*_4_ = *s*/2. Substituting the result of Equation (21) into Equation (8) gives Equation (22), which shows the magnitude of the radiant flux as *θ* → 0 is dependent on *k* and Δ*d* and independent of *y*:

(22) $$\Phi_{e\left(\theta \to 0\right)}=2\left(\frac{n_{RI}\epsilon_{0}c}{2}\right){E_{0}}^{2}z\cdot s(\cos(k2\Delta d)+1)$$

Note Equations (10) and (22) are equal, resulting in maximum radiant flux for translations where 2Δ*d* = *nλ*:

(23) $$\Phi_{e\left(\theta \to 0,2\Delta d=n\lambda \right)}=2\left(\frac{n_{RI}\epsilon_{0}c}{2}\right){E_{0}}^{2}z\cdot 2s$$

To work out the value of radiant flux for all other values of *θ_np_*, *i.e.*, *θ*_1,2,3,…_, substitute *θ_np_* = *n_p_λ/s* from Equation (16) into Equation (20), which renders:

(24) $$\Phi_{e\left(x_{1},x_{2}\right)}\propto s$$

Repeating the above for Φ*_e_* (*x*_3_, *x*_4_) again yields the same result, therefore the radiant flux at *primary node* angles *θ_ns_* = *θ*_1,2,3,.._ is:

(25) $$\Phi_{e\left(\theta_{1,2,3,\ldots }\right)}=2\left(\frac{n_{RI}\epsilon_{0}c}{2}\right){E_{0}}^{2}z\cdot s$$

Equation (25) also shows that the radiant flux at *θ_ns_*= *θ*_1,2,3,.._ is half maximum (*cf.* Equation (23)), and in contrast to the radiant flux at *θ_ns_* = *θ*_0_ given in Equation (22), *Φ_e_*_(_*_θ_*_1,2,3,..)_ is independent of *k* and Δ*d*. The reason for this is the width *s* of the active area is an integer multiple of the fringe line spacing at *primary node* angles *θ_np_* = *θ*_1,2,3,…_

When there is an exact multiple of fringe lines within the active area [18], the radiant flux across the active area is the mean of the maximum constructive and destructive interferences, *i.e.*, 50%. This means that if the active area is moved in either direction along the *x*-axis, the radiant flux remains static at 0.5 normalised magnitude. If mirror M2 translates, there will be no change in the radiant flux despite the fringe pattern moving back and forth across the active area.

### 2.4. Effect of Distance x and y of Photodetector from Origin with Varying θ

To determine the effect of distance *y* of the photodetector from the origin with varying *θ*, the position and slope of the fringe lines needs to be determined. This is done by finding the instances of maximum value of irradiance in Equation (7), *i.e.*, when:

(26) cos(k(y−2Δd−ycosθ−xsinθ))=1

Equation (26) is true when:

(27) $$k\left(y-2\Delta d-y\cos\theta -x\sin\theta \right)=2n_{f}\pi$$

Where *n_f_* is the *n^th^* fringe line. Solving for *y* gives:

(28) $$y=\frac{x\sin\theta }{\left(1-\cos\theta \right)}+\frac{n_{f}\lambda +2\Delta d}{\left(1-\cos\theta \right)}$$

Equation (28) defines the profile of the fringe pattern in the *x-y* plane as illustrated in Figure 2, where sin *θ*/(1 – cos*θ*) is the slope of the fringe lines and (*n_f_λ* + 2Δ*d*)/(1 – cos*θ*) is the *y* intercept. As only small angles are being considered it becomes indeterminate at *θ* = 0, which stands to reason as the fringe pattern is uniform across the active area as well as the *x-y* plane and no fringe lines are present.

By solving Equation (28) for *n_f_*, the number of fringes lines passing over a given point (*x*, *y*) can be calculated for *θ* increasing or decreasing from zero to a given wave front angle:

(29) $$n_{f}=\frac{y\left(1-\cos\theta \right)}{\lambda }-\frac{x\sin\theta }{\lambda }-\frac{2\Delta d}{\lambda }$$

The *y* term in the above equation is positive for *θ* ≠ 0 and is symmetrical in shape as a function of *θ*. For small angles sin *θ* = *θ*, therefore the coefficient of *x* is a linear function of angle *θ*. The polarity of *n_f_*, is therefore dependent on the magnitude and sign of *x*, *θ* and Δ*d*.

*n_f_* is the number of fringes counted at a point (*x*, *y*) as *θ* is varied. Assume Δ*d* = 0, there is a special case in Equation (29) when:

(30) $$\frac{y\left(1-\cos\theta \right)}{\lambda }=\frac{x\sin\theta }{\lambda }$$

and the fringe count *n_f_* = 0 despite *θ* > 0. In this case, fringe lines would have moved over point (*x*, *y*) in one direction as *θ* is increased and then back again as *θ* is increased further to the angle *θ* that satisfies Equation (30).

As discussed with Equation (28), the slope of the fringe lines is given by:

(31) $$\frac{y}{x}=\frac{\sin\theta }{\left(1-\cos\theta \right)}=\cot\left(\frac{\theta }{2}\right)$$

Where *θ*/2 is the angle of the normal of mirror M2 relative to the *y*-axis (Figure 2). Therefore, there is a set of points (*x*, *y*) coincident with the mirror normal that renders *n_f_* = 0.

### 2.5. Deriving the Speed of Fringe Movement

If Δ*d* is considered static (Δ*d* = 0), Equation (29) reduces to:

(32) $$n_{f}=\frac{y\left(1-\cos\theta \right)}{\lambda }-\frac{x\sin\theta }{\lambda }$$

Taking the derivative of the *x*-term delivers the speed of contraction/expansion of the fringe lines at the point (*x*, *y*):

(33) $$\frac{dn_{f}}{d\sin\theta }=-\frac{x}{\lambda }$$

The speed of sideways deflection of the fringe lines at point (*x*, *y*) results from calculating the derivative of the *y*-term:

(34) $$\frac{dn_{f}}{d\sin\theta }=\frac{y}{\lambda }\frac{\sin\theta }{\sqrt{1-\sin^{2}\theta }}=\frac{y}{\lambda }\tan\theta$$

The overall fringe movement speed is therefore:

(35) $$\frac{dn_{f}}{d\sin\theta }=-\frac{x}{\lambda }+\frac{y}{\lambda }\tan\theta$$

From Equation (35), the direction of fringe movement reverses if:

(36) $$-\frac{x}{\lambda }+\frac{y}{\lambda }\tan\theta =0$$

and therefore the angle of movement reversal *θ_rev_* is:

(37) $$\theta_{\text{rev}}=\tan^{-1}\frac{x}{y}=\sin^{-1}\frac{x}{\sqrt{x^{2}+y^{2}}}$$

That is, when the perpendicular of the wave front from the origin points towards +*x*. The mirror tilt angle is therefore *θ_rev_*/2. If *y* → 0, *θ_rev_* → *π*/2, *i.e.*, the smaller *y*, the larger is *θ_rev_*.

From Equation (29), the fringe count returns to zero if the numerical value of *x*- and *y*-terms cancel each other out, *i.e.*,:

(38) y−ycosθ−xsinθ=0

Which has solution:

(39) $$\sin\theta =\frac{2xy}{\left(x^{2}+y^{2}\right)}$$

This occurs at the angle *θ_n_*_= 0_:

(40) $$\theta_{n=0}=\sin^{-1}\frac{2xy}{\left(x^{2}+y^{2}\right)}$$

If *x* = *y*, then *θ_n_*_= 0_ = *π*/2, if *x* > *y*, then *θ_n_*_= 0_ > *π*/2 and conversely, if *y* > *x*, then *θ_n_*_= 0_ < *π*/2.

The relationship between *θ_n_*_= 0_ and *θ_rev_*, results in the identity of *θ_n_*_= 0_ ≡ 2*θ_rev_*.

From Equations (37) and (40):

(41) $$\theta_{\text{rev}}=\frac{1}{2}\sin^{-1}\frac{2xy}{\left(x^{2}+y^{2}\right)}=\sin^{-1}\frac{x}{\sqrt{x^{2}+y^{2}}}$$

Resulting in:

(42) $$\sin2\theta_{\text{rev}}=2\sin\theta_{\text{rev}}\cos\theta_{\text{rev}}=\frac{2xy}{\left(x^{2}+y^{2}\right)}$$

and proving the identity of *θ_n_*_= 0_ ≡ 2*θ_rev_* being correct.

### 2.6. Determination of the Damping Function of the Radiant Flux Curve

From Figure 3 it is evident that the normalised radiant flux curve oscillates about a level of 0.5 radiant flux and decreases its wave amplitude about this level with increasing wave front angle *θ*. As this behaviour constitutes a damped function, the radiant flux decay function is derived subsequently.

For a centred photodetector of width *s* and positioned at *x* = 0, with integral boundaries *x*_2_ = +*s*/2 and *x*_1_ = −*s*/2, *y* = 0 and Δ*d* = 0, the radiant flux *Φ_e_*_(_*_x_*_1_,*_x_*_2)_ in Equation (8) reduces to:

(43) $$\Phi_{e\left(x_{1},x_{2}\right)}\propto \frac{2\sin\left(\frac{ks\sin\theta }{2}\right)}{k\sin\theta }+s$$

In Equation (43), the maximal radiant flux corresponds to 2s (*cf.* Equations (11) and (23): radiant flux = constant * *z* * 2*s; z* is considered unity as the *z*-direction is perpendicular to fringe lines).

Normalising the radiant flux to 2*s* delivers the normalised radiant flux Φ*n*:

(44) $$\Phi_{n}\propto \frac{\sin\left(\frac{ks\sin\theta }{2}\right)}{sk\sin\theta }+\frac{1}{2}$$

As the normalised radiant flux ranges from 0 to +1, it is converted to a range from −1 to +1 in order to compare it to a standard sine function:

(45) $$\Phi_{n}\propto 2\frac{\sin\left(\frac{ks\sin\theta }{2}\right)}{sk\sin\theta }=\frac{\sin\left(\frac{ks\sin\theta }{2}\right)}{\frac{ks\sin\theta }{2}}=\text{sinc}\left(\frac{ks\sin\theta }{2}\right)\approx \text{sinc}\left(\frac{ks\theta }{2}\right)$$

for small *θ*. Considering that *k* = 2*π*/*λ* and that a damped function (e.g., cardinal sine) corresponds to an undamped function times a decay function, the decay function *F_D_* is identical to the reciprocal of the denominator (*ks* sin *θ*)/2 in Equation (45). For small *θ*:

(46) $$F_{D}=\frac{2}{sk}\cdot \frac{1}{\theta }=\frac{\lambda }{\pi s}\cdot \frac{1}{\theta }$$

Equation (46) is the constitutive equation of the radiant flux decay function at *x* = 0 and *y* = 0. *F_D_* is a reciprocal function of *θ*, with a decay constant *C_D_* of $\frac{2}{sk}$or $\frac{\lambda }{\pi s}$.

## 3. Results

The radiant flux and the fringe count are influenced by a range of parameters, namely the wave front angle *θ*, the position *x* of the photodetector with respect to the centre line, the laser wave length *λ*, the distance *y* between the mirror and the photodetector, and the side length *s*, *i.e.*, the size of the photodetector, all of which are variable. The influence of these parameters is explained in a systematic way based on the equations derived in the Mathematical Analysis section.

### 3.1. Influence of θ on the Radiant Flux

The effect of *θ* on the magnitude of the radiant flux also depends on the values of the other abovementioned variable parameters. In order to demonstrate this, the radiant flux is examined with four different conditions, in which some parameters are kept constant whereas others are variable.

#### 3.1.1. *θ* = Variable, *x* = 0, *y* = 0, *s* = Variable

**Figure 4.**
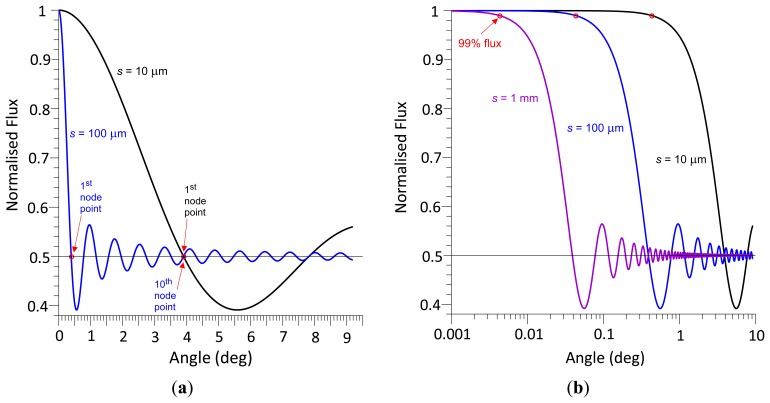
Normalised radiant flux against wave front angle; (a) node points; (b) wave front angle at 99% radiant flux; *s* = 10 μm, 100 μm, and 1 mm; *λ* = 680 nm.

Figure 4a shows the radiant flux curves of two different photodetector areas. If *θ* = 0, the radiant flux is 100%. With increasing *θ*, the radiant flux decreases first, reaches the first *primary node* at a radiant flux magnitude of 50% and oscillates about the 50% level with decreasing radiant flux amplitude. The normalised radiant flux curve corresponds to a damped sine function with a damping function of 2/(*skθ*) or *λ*/(*πsθ*). Multiplying a sine function of the form sin (*πsθ*/*λ*), where *λ/s* represents the reciprocal value of the first node angle *θ_np_*, delivers a damped sine wave, which, after adding 1 and dividing the sum by 2, results in the normalised radiant flux curve. In contrast to a standard sine wave, where the first minimum is at (3/2) π, *i.e.*, at 1.5 *θ_np_*, the non-linear decay rate causes the first minimum to be located at an angle of 1.4304 *θ_np_*, with a normalised radiant flux magnitude of 0.3913832. This magnitude is independent of *s* and *λ*.

**Figure 5.**
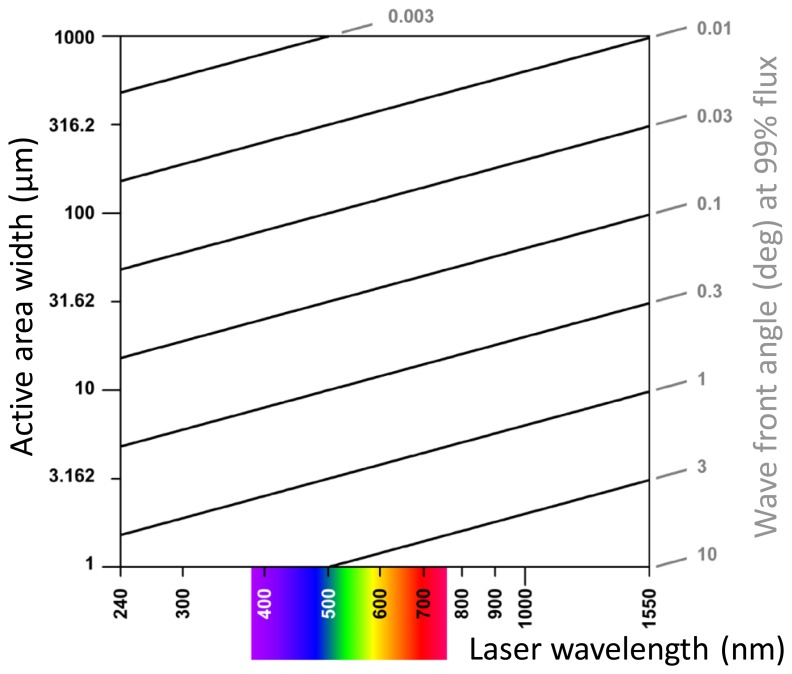
Contour plot of equal wave front angles of 99% radiant flux as a function of the side length *s* of a square photodetector area and laser wave length *λ*.

According to Equation (16), the smaller *s*, the larger is the wave front angle at the first node point. If *s* = 100 μm and *λ* = 680 nm, the first and tenth *primary nodes* are at *θ* = 0.3896° and 3.896°, respectively, and if *s* = 10 μm, the first *primary node* is at 3.896° (Figure 4a). The smaller is *s*, the slower the radiant flux decreases with increasing wave front angle. If *s* = 10 μm, 100 μm, and 1 mm, the angle at 99% radiant flux is located at *θ* = 0.43°, 0.043°, and 0.0043° (Figure 4b), respectively. According to Equation (16)*s* and *λ* have opposite effects: reducing *s* by a factor of two results in the same angles of *primary node* points and 99% radiant flux as does a two-fold increase of *λ*. Figure 5 exemplifies this principle in a contour plot of equal angles of 99% radiant flux as a function of *s* and *λ*.

At this point it has to be mentioned that the same radiant flux curves are obtained if *y* > 0 and *x* = *y*(1/sin*θ* – 1/tan*θ*) according to Equation (31). Equation (31) provides the solution for *n_f_* = 0, which deviates in *x*-direction if *y* > 0. This is explained in more detail below in the section dealing with influence on fringe count.

#### 3.1.2. *θ* = Variable, *x* = Variable, *y* = 0, *s* = Variable

**Figure 6.**
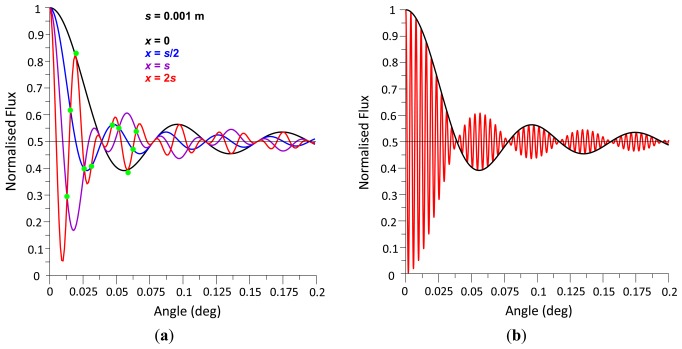
Normalised radiant flux against wave front angle at *x* = 0, *s*/2, *s*, 2*s* (a); and *x* = 0 and 10*s* (b); *s* = 0.001, *λ* = 680 nm; *secondary nodes* are marked with green dots in the first cycle up to the 2nd node point (a).

**Figure 7.**
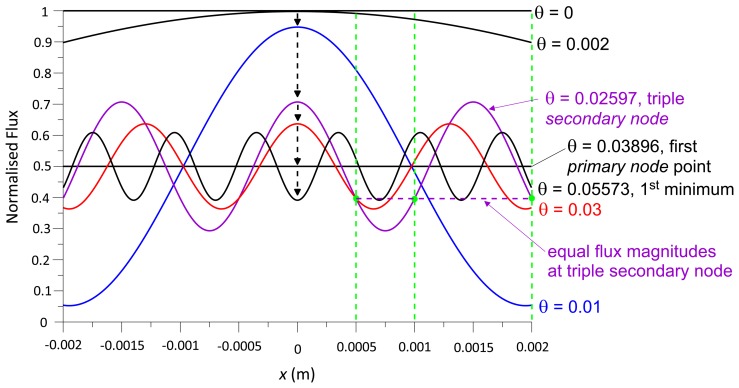
Normalised radiant flux across an *x*-range of 4 mm at different wave front angles *θ* (in degrees); the dashed green lines indicate the *x*-position of the blue radiant flux curve shown in Figure 6a (*s* = 1 mm, *x* = *s*/2, *s*, 2*s*); the dashed purple line indicates the radiant flux level (intersections of green dashed lines and purple radiant flux curve) at the first triple *secondary node* (at *θ* = 0.02597°); the 1st minimum refers to the radiant flux curve at *x* = 0 (Figure 6a).

Photodetector positions *x* ≠ 0 changes the curves shown in Figure 4a insofar as the number of 50% radiant flux transitions is larger than the number of *primary node* points. The larger *x*, the more the radiant flux curve oscillates between the node points. In Figure 6, the radiant flux curve at *x* = *s*/2 intersects the 0.5 radiant flux level once between each pair of node points, the curve at *x* = *s* does so twice, at *x* = 2*s* four times and at *x* = 10*s* twenty times. The larger *x*, the higher is the density of the radiant flux curve filling up the area under the radiant flux curve at *x* = 0 (Figure 6b), which acts like an envelope curve for radiant flux oscillations at larger *x*. This is insofar important to note as it shows that the modulation amplitude of the radiant flux across *x* (Figure 7), is unaffected by *x*.

Figure 6a shows *secondary node* points, *i.e.*, intersections of the two curves at radiant flux magnitudes other than 50%. Independent of the position *x*, all curves intersect at the *primary node* points. The radiant flux at the *primary node* points is constant (50%), whereas the radiant flux at the *secondary nodes* is variable and a function of *x*. For example, at multiples of 0.02597° (Figure 6a), the three radiant flux curves of *x* = *s*/2, *s* and 2*s*, with *s* = 0.001, intersect; at the 3rd and 6th intersection, the *secondary nodes* are identical to the *primary nodes* (2nd and 4th).

As the angle *θ* increases, so does the number of fringe lines per unit *x* (Figure 7). At *θ* = 0, the radiant flux is constant at 100%. After a slight increase in *θ*, the radiant flux oscillates between 100% and 0%, *i.e.*, the maximal radiant flux is still very close to 100% (Figure 4b). Further increase in *θ* reduces the radiant flux amplitude, which fluctuates about 50% until the modulation amplitude converges to 0 at the 1st node point and remains constant at 50% radiant flux. Further increase in *θ* expands the modulation amplitude, however, the polarity of the radiant flux curve changes, *i.e.*, peaks at *x* = 0 before the node point are converted to troughs after the node point.

#### 3.1.3. *θ* = Variable, *x* = 0, *y* = Variable, *s* = Variable

**Figure 8.**
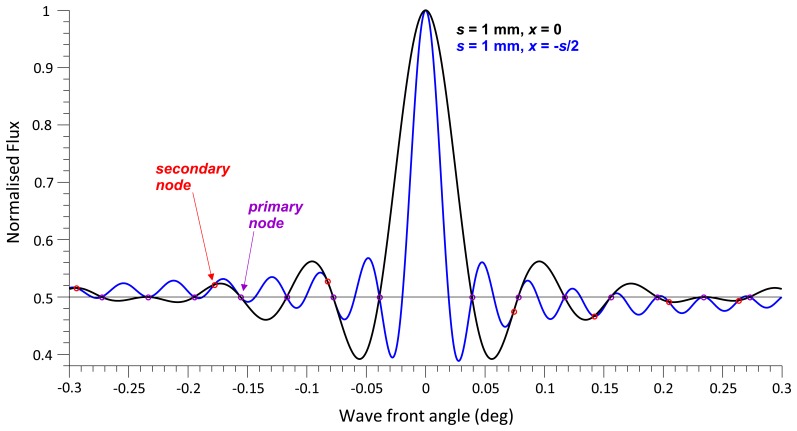
Normalised radiant flux against wave front angle at two different *x*; *λ* = 680 nm, *y* = 0.02 m.

When introducing the distance *y* between the plane of the photodetector and the tilting mirror, the radiant flux curve can be entirely below or above the 50% radiant flux level, touching it only at the *primary node* points (Figure 8). The radiant flux curve is then superimposed by a further oscillation of a longer wave length. At the 6th *primary node* point of *θ* = 0.23377°, the radiant flux curve does not cross the 50% radiant flux level; nevertheless, the polarity changes in the same way as shown in Figure 7. The distance *y* does not affect the *primary node* points according to Equation (16), whereas the *secondary nodes* are a function of *y* (as well as *s* and *θ*).

**Figure 9.**
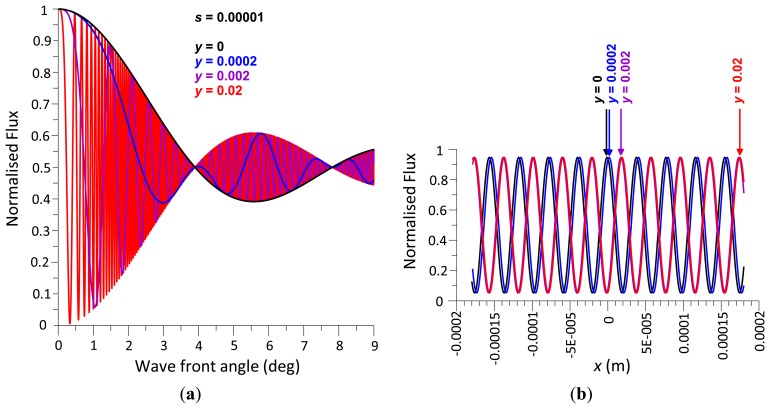
Normalised radiant flux against wave front angle (a) and *x* (b); *s* = 0.00001, *y* = 0, 0.0002, 0.002, and 0.02 m; (b) shows the position of the centre fringe (*i.e.*, fringe number 0) radiant flux at angle *θ* = +1° and movement of the fringe pattern with increasing *y* (note that amplitude range and fringe density are independent of *y*).

Increasing *y* (Figure 9) has the same effect as increasing *x* (Figure 6): the radiant flux curve oscillates more frequently under the envelope of the radiant flux at *y* = 0 (Figure 9a). This does not affect the modulation amplitude of the radiant flux (Figure 9b), which remains the same across *x* at a specific angle *θ*, however, the centre fringe line is more deflected off-centre with *θ*, the larger is *y* (Figure 9b).

#### 3.1.4. *θ* = Variable, x = Variable, y = Variable, s = Variable

**Figure 10.**
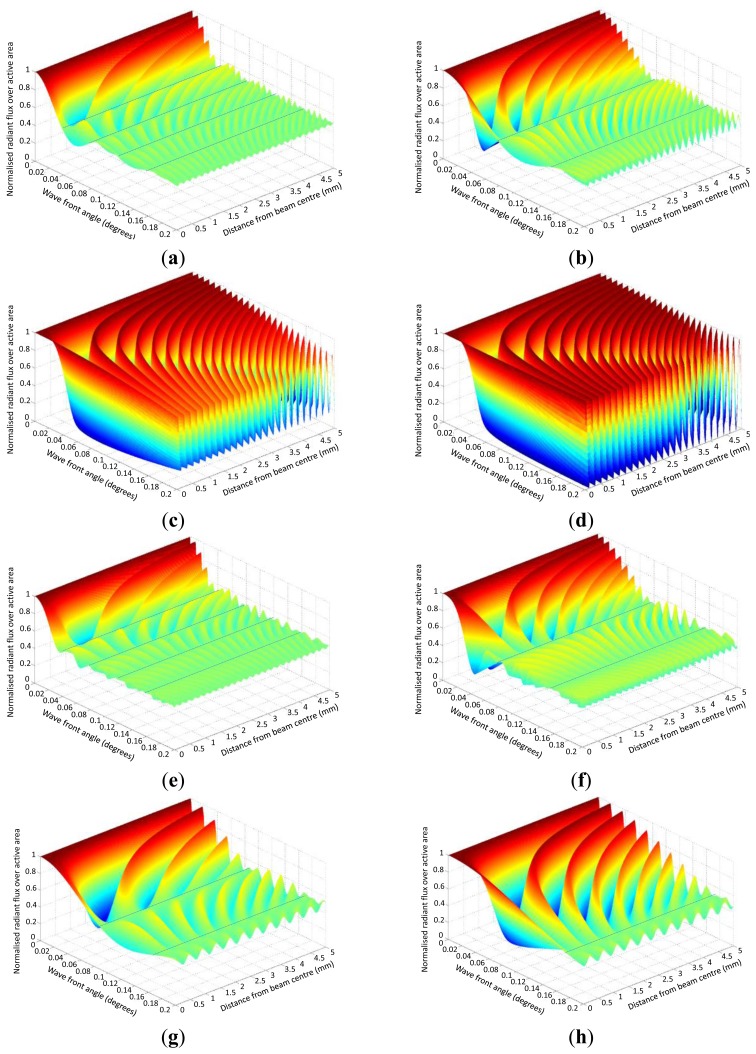
Normalised radiant flux against wave front angle *θ* = 0° … 0.2°) and distance from the beam centre (*x* = 0 mm … 5 mm) at *y* = 20 mm, and *λ* = 680 nm; (a): *s* = 1 mm & *y* = 20 mm, *λ* = 680 nm; (b): *s* = 0.5 mm & *y* = 20 mm, *λ* = 680 nm; (c): *s* = 0.1 mm & *y* = 20 mm, *λ* = 680 nm; (d): *s* = 0.01 mm & *y* = 20 mm, *λ* = 680 nm; (e): *s* = 1 mm & *y* = 1 m, *λ* = 680 nm; (f): *s* = 0.5 mm & *y* = 1 m, *λ* = 680 nm; (g): *s* = 1 mm & *y* = 1 m, *λ* = 1,550 nm; (h): *s* = 0.5 mm & *y* = 1 m, *λ* = 1,550 nm.

Figure 10 summarises the influence of *θ*, *s*, *x*, *y* and *λ* on the normalised radiant flux. The difference between Figure 10a–d is that the radiant flux before the first *primary node* point decays slower the smaller *s* is. Figure 10d shows for small angles that the modulation amplitude remains constant across *x* and *θ*.

Figure 10e,f shows with greater *y*, the more the centre fringe line deflects towards larger positive *x* and that fringe lines from the negative *x*-side cross over to the positive-side.

The dotted lines in Figure 10a,b,e,f shows the *primary nodes* for *s* = 1 mm, *s* = 0.5 mm and that the *primary nodes* are dependent on *s* and independent of *y* for constant *λ*.

Figure 10g,h shows with greater *λ* for equivalent *s* (*cf.*Figure 10a,b) that the interval of *primary node* angles is greater. Also, the radiant flux decays slower before the 1st *primary node* for greater *λ*.

Figure 10g,h shows with greater *λ* for equivalent *s* (*cf.* Figure 10a,b) that the radiant flux drops off slower before the 1st *primary node*.

Figure 10 shows at *θ* = 0°, the normalised radiant flux = 1 and is independent of *x*, *s*, *y* and *λ*.

### 3.2. Influence of x and y on the Fringe Count

The number of fringe lines *n_f_* passing over a point (*x*, *y*) within the fringe pattern is given by Equation (29), which is a function of the fringe lines tilting (*y* term), the fringe lines contracting/expanding (*x*-term) and moving mirror displacement (Δ*d*).

Assuming Δ*d* = 0, as the moving mirror tilts from orthogonality, fringe lines are produced with a slope that is parallel to the normal of the mirror (Equation (31) and Figure 2). The *y*-term in Equation (29) is linked to the slope of the fringe lines, *i.e.*, as the wave front angle increases then so does the fringe lines (in cross-section). With *x* and *θ* static, the further the point is up the *y*-axis, the greater the number of fringe lines that tilt across the point. The angle *θ* between the two wave fronts can be positive or negative with respect to the *y*-axis, however, the coefficient of the *y*-term is always positive and therefore only has an additive effect on *n_f_*.

The *x*-term of Equation (29) is linked to contraction/expansion of the fringe lines with wave front angle. The focal point of the contraction/expansion is the normal of the mirror that is coincident with the axis of tilt.

With Δ*d* = 0, as *θ* is increased a central fringe line is generated and aligns itself with this normal. Fringe lines develop to the left and right of this normal and contract toward it. For a given point (*x*, *y*) in the fringe pattern, as *θ* is increased, more and more fringe lines will develop and cross over the point. As the central fringe line is essentially static, fringe lines to the far left and right move far quicker than those closer to the central fringe line.

**Figure 11.**
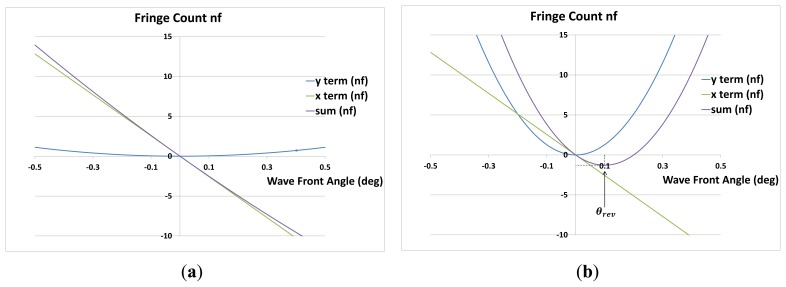
The effect of variable *y* on the fringe count; (a) *x* = 0.001 m, *y* = 0.02 m, *λ* = 680 nm, Δ*d* = 0; (b) *x* = 0.001 m, *y* = 0.573 m, *λ* = 680 nm, Δ*d* = 0.

Figure 11 and Equation (33) confirm this behaviour showing that the speed of contraction/expansion (from the *x*-term of Equation (32)) of the fringe pattern is a constant. Therefore an active area located further from the centre of the beam will experience more fringe lines passing over it than one located closer to the centre when mirror M2 tilts. Parameters *x* and *λ* have opposite effects on the contraction/expansion speed.

The speed of the fringe line tilt (from the *y*-term of Equation (32)) results from Equation (34). This fringe tilt speed is a tangent function of the wave front angle, independent of *x*, *i.e.*, of the lateral position of the photodetector, but dependent on *y* and *λ*. The speed of fringe line tilt is initially smaller than the speed of contraction, as the former is zero if *θ* = 0 (tan *θ* = 0).

**Figure 12.**
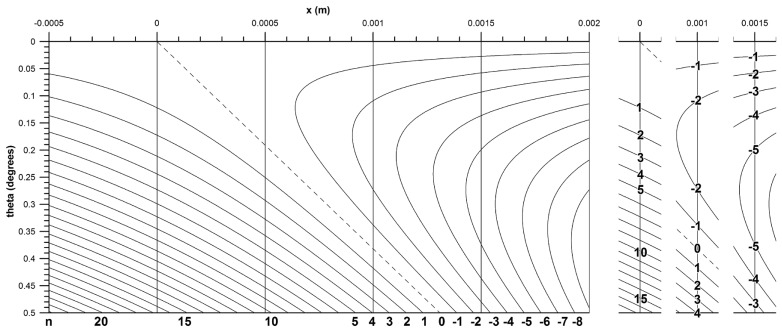
Fringe counting as a function of *x* and *θ; x* = 0.001 m, *y* = 0.30 m, *λ* = 680 nm, Δ*d* = 0; the fringe count is shown on the right side, for *x* = 0, 0.001, and 0.0015 m; “0” = peak of centre fringe; positive and negative fringe numbers refer to negative and positive *x*, respectively, *i.e.*, to left and right sides of the centre fringe line “0”.

Figure 12 shows the effect of increasing and then decreasing fringe numbers with progressive wave front angle. The fringe count on the positive *x*-side are acutely curved initially, the speed of the centre fringe line deflection lags behind the speed of contraction. Subsequently, the former speed term catches up and eventually overtakes the latter term. This results in the fringe lines initially moving over an off-centre photodetector in one direction and then moving over the same detector again but in opposite direction, thereby first increasing the fringe count and subsequently decreasing it. Figure 12 also shows that the larger *x*, the larger is *θ_rev_*.

If Δ*d* is dynamic and *θ* is variable, then the fringe count is affected by transition of the fringe lines across the photodetector due to mirror translation in addition to fringe tilt and fringe contraction/expansion. The direction of transition of the fringe lines is dependent on whether translation of the mirror is in the positive or negative *y* direction.

The effect of fringe tilt and *y* position of the photodetector can also be shown taking the photodetector parameters given in Figure 11d where *s* = 0.01 mm, *y* = 20 mm, *λ* = 680 nm and centering the photodetector on the *y* axis (*i.e.*, *x* = 0 mm). From Equation (8) the normalised radiant flux is calculated to be 0.997 for a wave front angle *θ* of 0.05°. Relocating the photodetector at *y* = 100 mm returns a normalized flux of 0.938, which is reduced from the first location as a result of fringe tilt. To work out what this change in radiant flux represents in terms of change in fringe position, substitute each flux value into the equation Φ = *Φ*_0_*sin*(*kx*′), where Φ_0_ = 1 is the maximum flux amplitude, *k* = 2*π*/*d_f_*, *d_f_* = *λ*/*θ* is the fringe width and *x*′ represents the first and second position respectively of the fringe lines. Subtracting the two equations and solving for the change in fringe position gives 34.64 μm. Fringe width is 779.2 μm, therefore the change in fringe position due to the photodetector being located further away represents a phase difference of 16° and consequently represents a difference in *n_f_* between the two positions with varying *θ*.

## 4. Discussion

The focus of this paper has been to establish the behaviour of the radiant flux of the interferogram over a photodetector of rectangular aperture that is variable; in size; in displacement across the interferogram; and, in axial distance from the source of interference, for variable angle between the two wave fronts and variable wavelength.

The most apparent observation from the mathematical analysis in this study is that the radiant flux decays rapidly with increasing wave front angle with the recurrence of *primary nodes* where the radiant flux decays to 50% maximum and the modulation amplitude reduces to zero. This observation is also confirmed by [3,5–10,17] where radiant flux is calculated over a disc and also by [8,11] where the radiant flux is calculated over a square area. The modulation amplitude in this study and the literature is found to decay as a cardinal sine function. However, this study has gone further to determine that the radiant flux decays with a decay function that is a reciprocal function of the wave front angle with decay constant that is proportional to the wavelength and is inversely proportional to the photodetector active area width.

What is so far not apparent from the literature, nor is it evident in the figures, is that between each *primary node* the polarity of the radiant flux reverses. This phenomenon has an adverse effect on phase measurement accuracy where the wave front angle has been allowed to increase beyond a *primary node* and the modulation amplitude is sufficiently large enough for fringes to be counted.

In the literature [3,5–11,17], the boundary of the radiant flux calculation is centred on the interferogram, giving just a single radiant flux curve decaying as a cardinal sine function. Whereas, in this study, the radiant flux boundary is derived to be variable across the interferogram. This variant shows that as the boundary is moved off centre, the radiant flux oscillates increasingly about the 50% normalised maximum as an integer multiple of the distance from centre. Additionally, the amplitude of the oscillation is bounded by the centred radiant flux curve.

A further finding from this study, which to the best of our knowledge, is not mentioned in the literature is how the radiant flux is affected when the fringe lines tilt and contract/expand with varying wave front angle. Having included as an integral parameter the axial distance of the photodetector from the interferometer, it is found for a centred photodetector that fringe tilt initially lags fringe contraction, but then fringe tilt becomes increasingly dominant on the radiant flux with increasing distance of the photodetector from the beamsplitter.

Interferometry applications that use a plane flat mirror with translation stage, and discrete photodetectors [12–15] or position sensitive device [16] will suffer erroneous measurements if the wave front angle is not limited to give acceptable modulation amplitude. This can be done by choosing an appropriate photodetector aperture width that is an order of magnitude less than the fringe line spacing. Increasing the wavelength improves modulation amplitude for equivalent photodetector aperture widths.

If the wave front angle varies beyond *primary node* angles the radiant flux reverses polarity introducing an additional error with a *π* radian phase change in photodetector output.

Alignment of the photodetector with the centre of the interferogram reduces the susceptibility of the fringe lines crossing over the photodetector as they contract as a linear function of distance from centre with increasing wave front angle.

Finally, the tilt angle of the fringe lines increases with increasing wave front angle and they cross over the central axis of the interference beam with increasing distance from the beamsplitter. Therefore to limit the error in measurement that this produces, the photodetector should be located as close as possible to the beamsplitter.

## 5. Conclusions

The radiant flux Φ across the active area of a photodetector is a damped sine function (*i.e.*, a cardinal sine function) of the wave front angle *θ*, with a reciprocal decay of the ratio of wavelength *λ* to detector width *s*. The larger *s* and the smaller *λ*, the faster is the decay of the radiant flux.

If the radiant flux magnitude at wavefront angle *θ* = 0 is normalised to 100%, then the radiant flux magnitude at the *primary node* points is 50% and at the first minimum is 39.14%. The polarity of the radiant flux changes exclusively at every *primary node* point. The radiant flux magnitude at specific *θ* is independent of any parameter if the centre of the fringe beam coincides with the centre of the photodetector.

The larger the distance *x* of the photodetector from the centre of the fringe beam and/or the distance *y* between mirror and photodetector, the more the radiant flux oscillates under the envelope radiant flux curve generated if *x* and *y* = 0. These two parameters do not affect the modulation amplitude of the radiant flux.

The movement of fringe lines with increasing *θ* is a combined effect of fringe contraction (*x*-dependent; the faster the more the detector is off centre) and fringe tilt (*y*-dependent; the faster the larger *y*). Fringe contraction and tilt movement can have opposite effects, with fringe contraction lagging behind fringe tilt, such that the fringe count first increases, then decreases and then returns to zero.

Consequently, significant fringe count errors occur if the photodetector is operated near or beyond *primary nodes* where radiant flux modulation reduces to zero and then changes polarity, or if the *x* and *y* distances of the photodetector are large therefore increasing the fringe contraction and fringe tilt influence.

## Appendix

This appendix provides a detailed derivation of the equations in the paper “Theoretical Analysis of Interferometer Wave Front Tilt and Fringe Radiant Flux on a Rectangular Photodetector” by authors R.M. Smith and F.K. Fuss.

The equation numbers in this appendix follows the equation numbering in the parent paper, however, where additional equations are included they are designated an alphanumeric reference.

### A1. Derivation of the Equation for Radiant Flux

The electric field of a plane wave is given by Equation (A1)

(A1) $$E\left(r,t\right)=E_{0}e^{i(k.r-\omega t)}$$

where **E** is the time (*t*) dependent electric field, **r** is the unit vector of the electric field in 3 dimensional space, *i.e.*, **r** = *x***x̂** + *y***ŷ** + *z***ẑ** and **x̂**, **ŷ** and **ẑ** are unit vectors along the *x*, *y* and *z* axes, **E_0_** is the vector amplitude of the wave, **k** is the wave vector where **k** = k**u**, where **u** is the unit vector defining the direction of propagation and $\left|\text{k}\right|=k=\frac{2\pi }{\lambda }$, *λ* is the wavelength of the light source, *k* is the wave number and *ω* is the angular frequency of the wave.

Figure 2 depicts the linear optical equivalent of the Michelson interferometer with the virtual source wave front approaching mirrors M1 and M2 from the top of the figure. With reference to the origin, the reflected wave fronts 1 and 2 from respective mirrors have wave vectors **k_1_** and **k_2_**.

(A2) $${\text{k}}_{1}=k\hat{y}$$

(A3) $$k_{2}=k\sin\theta \hat{x}+k\cos\theta \hat{y}$$

Also depicted in Figure 2, the source wave front travels a distance Δ*d* further to M1 before it is reflected. Therefore, the optical path difference (OPD) between wave fronts 1 and 2 is 2Δ*d* resulting in wave front 1 having a phase lag of *k*2Δ*d* relative to wave front 2.

The sum of the electric fields of wave fronts 1 and 2 is therefore

(A4) $$E_{\text{sum}}\left(r,t\right)=E_{0}e^{i\left(k_{1}\cdot r-k2\Delta d-\omega t\right)}+E_{0}e^{i\left(k_{2}\cdot r-\omega t\right)}$$

(A5) $$\begin{array}{ll} E_{\text{sum}}\left(x,y,z\right) & =E_{0}e^{i\left(k\left(y-2\Delta d\right)-\omega t\right)}+E_{0}e^{i\left(k\left(y\cos\theta +x\sin\theta \right)-\omega t\right)}\\ & =E_{0}e^{-i\omega t}\left(e^{ik\left(y-2\Delta d\right)}+e^{i\left(k\left(y\cos\theta +x\sin\theta \right)\right)}\right) \end{array}$$

The irradiance *I* of an electric field is given by Equation (A6) and is the radiant flux of the electric field delivered per area to a given surface with units Wm^−2^. *i.e.*, radiant flux density

(A6) $$I=\left(\frac{n_{RI}\epsilon_{0}c}{2}\right)E_{\text{sum}}\cdot E_{\text{sum}}^{*}$$

Where *n_RI_* is the refractive index of the medium, *c* is the speed of light in vacuum, *ε*_0_ is the vacuum permittivity, and $E_{\text{sum}}^{*}$ the complex conjugate of *E_sum_*.

(A7) $$\begin{array}{ll} I & =\left(\frac{n_{RI}\epsilon_{0}c}{2}\right)E_{0}e^{-i\omega t}\left(e^{ik\left(y-2\Delta d\right)}+e^{ik\left(y\cos\theta +x\sin\theta \right)}\right)\cdot E_{0}e^{i\omega t}\left(e^{-ik\left(y-2\Delta d\right)}+e^{-ik\left(y\cos\theta +x\sin\theta \right)}\right)\\ & =\left(\frac{n_{RI}\epsilon_{0}c}{2}\right){E_{0}}^{2}\left(2+2\cos\left(k\left(y-2\Delta d-y\cos\theta -x\sin\theta \right)\right)\right)\\ & =2\left(\frac{n_{RI}\epsilon_{0}c}{2}\right){E_{0}}^{2}\left(1+\cos\left(k\left(y-2\Delta d-y\cos\theta -x\sin\theta \right)\right)\right) \end{array}$$

Equation (A7) indicates that the irradiance at a point (*x, y*) in the fringe pattern created by wave fronts 1 and 2 is dependent on the values of *x* and *y*, wave number *k*, which is a function of wavelength, the angle *θ* between the wave fronts and the optical path difference 2Δ*d*.

If Equation (A7) is integrated along the *x*-axis between arbitrary points *x*_1_ and *x*_2_ and then multiplied by side length *z* in the *z* -direction to create an area across the photodetector, the solution is the radiant flux incident on a rectangle of side lengths *x*_2_ –*x*_1_ = *s* and *z*. As mirror M2 is only tilted about the *z*-axis, variable *z* does not need to be included in the integration as it behaves purely as a multiplier to the solution of the integration along the *x*-axis. Therefore:

(A8) $$\begin{array}{ll} \Phi_{e} & =z\cdot \int_{x}\text{I dx}=2\left(\frac{n_{RI}\epsilon_{0}c}{2}\right){E_{0}}^{2}z\int_{x_{1}}^{x_{2}}\left(1+\cos\left(k\left(y-2\Delta d-y\cos\theta -x\sin\theta \right)\right)\right)dx\\ & =2\left(\frac{n_{RI}\epsilon_{0}c}{2}\right){E_{0}}^{2}z\cdot {\left|\frac{1}{k\sin\theta }\left(\sin\left(k\left(x\sin\theta +y\cos\theta -y+2\Delta d\right)\right)+kx\sin\theta \right)\right|}_{x_{1}}^{x_{2}} \end{array}$$

The radiant flux Φ_e_ given by Equation (A8) is expressed in Watts (W) and is the total radiant power of the interference beam incident on the defined rectangular active area of the photodetector. At *θ* = 0, $\Phi_{e}=\frac{0}{0}$which is indeterminate, therefore applying L'Hôpital's rule to the integral solution of Equation (A8) for *θ* → 0 returns:

(A9) $$\begin{array}{l} \frac{\left(\sin\left(k\left(x\sin\theta +y\cos\theta -y+2\Delta d\right)\right)+kx\sin\theta \right)}{k\sin\theta }\\ =\underset{\theta \to 0}{\text{lim}}\frac{\left(\cos\left(k\left(x\sin\theta +y\cos\theta -y+2\Delta d\right)\right)\right)\cdot k\left(x\cos\theta -y\sin\theta \right)+kx\cos\theta }{k\cos\theta }\\ =\underset{\theta \to 0}{\text{lim}}\frac{\left(\cos\left(k2\Delta d\right)\right)\cdot kx+kx}{k}\\ =x\left(1+\cos\left(k2\Delta d\right)\right) \end{array}$$

Therefore, as *θ* → 0, the radiant flux derived in Equation (A8) tends to

(A10) $$\begin{array}{ll} \Phi_{e\left(\theta \to 0\right)} & =2\left(\frac{n_{RI}\epsilon_{0}c}{2}\right){E_{0}}^{2}z\cdot {\left|x\left(1+\cos\left(k2\Delta d\right)\right)\right|}_{x_{1}}^{x_{2}}\\ & =2\left(\frac{n_{RI}\epsilon_{0}c}{2}\right){E_{0}}^{2}z\cdot s\left(1+\cos\left(k2\Delta d\right)\right) \end{array}$$

It is worth noting that at *θ* = 0, the irradiance in Equation (A7) reduces to

(A10a) $$I_{\left(\theta =0\right)}=2\left(\frac{n_{RI}\epsilon_{0}c}{2}\right){E_{0}}^{2}\left(1+\cos\left(k2\Delta d\right)\right)$$

and differs with Equation (A10) by the area *z* · *s*.

Equation (A10) indicates that as *θ* → 0 the magnitude of the radiant flux is dependent on the area *z* · *s*, *k* and Δ*d* but is independent of *y*.

The irradiance and radiant flux in Equations (A10) and (A10a) are maximum when *cos*(*k*2Δ*d*) = 1, *i.e.*, when 2Δ*d* = *n_f_λ*, where *n_f_* is an integer equivalent to the number of fringe lines and 2Δ*d* is the optical path difference. Whenever the OPD is an integer multiple of the wavelength, the two wave fronts in Figure 2 are in phase with one another resulting in maximum radiant flux, *i.e.*,

(A11) $$\Phi_{e\left(\theta \to 0,2\Delta d=n_{f}\lambda \right)}=2\left(\frac{n_{RI}\epsilon_{0}c}{2}\right){E_{0}}^{2}z\cdot 2s$$

To demonstrate the behaviour of the radiant flux over differing integral boundaries, Figure 3 shows the radiant flux curves for two sets of integral boundaries that are equal in length with assigned variables defined as follows that have been substituted in Equation (A10); *y* = 0 m, *λ* = 680 × 10^−9^ m therefore *k* = 9239978, *Δd* = 0 m, integral width *s* = 0.001m, red curve integral boundaries *x*_2_ = 0.0005 m, *x*_1_ = −0.0005 m, blue curve integral boundaries *x*_2_ = 0.001 m, *x*_1_ = 0 m.

It can be seen from the Figure 3 what appears to be node points at half the normalised radiant flux that are cyclic, which have been termed *primary nodes*, and this phenomenon is explored further below. What is also noticeable is the two curves converge as *θ* → 0 as predicted in Equation (A10).

### A2. Identifying Specific Wave Front Angles θ_n_ With Invariable Φ**_e_** for Two Sets of Integral Boundaries

To analyse the effect of mirror tilt angle on two separate rectangular areas of equal size and determine the node points observed in Figure A1, consider only the integral solution of Equation (A8). If we define the two intervals along the *x*-axis with upper and lower limits *x*_1_, *x*_2_ & *x*_3_,*x*_4_ such that *x*_2_ – *x*_1_ = *x*_4_ – *x*_3_ = *s* and substitute in the Equation (A8) we get after simplification Equations (A12) and (A13):

(A12) $$\begin{array}{cc} \Phi_{e\left(x_{1},x_{2}\right)} & \\ & \propto \frac{1}{k\sin\theta }\left[\sin\left(k\left(x_{2}\sin\theta +y\cos\theta -y+2\Delta d\right)\right)+kx_{2}\sin\theta -\sin\left(k\left(x_{1}\sin\theta +y\cos\theta -y+2\Delta d\right)\right)-kx_{1}\sin\theta \right] \end{array}$$

(A13) $$\begin{array}{cc} \Phi_{e\left(x_{3},x_{4}\right)} & \\ & \propto \frac{1}{k\sin\theta }\left[\sin\left(k\left(x_{4}\sin\theta +y\cos\theta -y+2\Delta d\right)\right)+kx_{4}\sin\theta -\sin\left(k\left(x_{3}\sin\theta +y\cos\theta -y+2\Delta d\right)\right)-kx_{3}\sin\theta \right] \end{array}$$

To solve for *θ* and *x*, let Equation (A12) = Equation (A13)

(A13a) $$\begin{array}{l} \sin(k(x_{2}\sin\theta +y\cos\theta -y+2\Delta d))+kx_{2}\sin\theta -\sin(k(x_{1}\sin\theta +y\cos\theta -y+2\Delta d))-kx_{1}\sin\theta \\ =\sin\left(k\left(x_{4}\sin\theta +y\cos\theta -y+2\Delta d\right)\right)+kx_{4}\sin\theta -\sin\left(k\left(x_{3}\sin\theta +y\cos\theta -y+2\Delta d\right)\right)-kx_{3}\sin\theta \end{array}$$

(A13b) $$\begin{array}{l} \sin\left(k\left(x_{2}\sin\theta +y\cos\theta -y+2\Delta d\right)\right)-\sin\left(k\left(x_{1}\sin\theta +y\cos\theta -y+2\Delta d\right)\right)\\ =\sin\left(k\left(x_{4}\sin\theta +y\cos\theta -y+2\Delta d\right)\right)-\sin\left(k\left(x_{3}\sin\theta +y\cos\theta -y+2\Delta d\right)\right) \end{array}$$

Applying the identity below and simplifying Equation (A13b) yields Equation (A14).

(A13c) $$\sin A-\sin B=2\sin\left(\frac{A-B}{2}\right)\cos\left(\frac{A+B}{2}\right)$$

(A13d) $$\begin{array}{c} 2\sin\left(\frac{k\left(\left(x_{2}\sin\theta +y\cos\theta -y+2\Delta d\right)-\left(x_{1}\sin\theta +y\cos\theta -y+2\Delta d\right)\right)}{2}\right)\\ \cos\left(\frac{k\left(\left(x_{2}\sin\theta +y\cos\theta -y+2\Delta d\right)+\left(x_{1}\sin\theta +y\cos\theta -y+2\Delta d\right)\right)}{2}\right)\\ =2\sin\left(\frac{k\left(\left(x_{4}\sin\theta +y\cos\theta -y+2\Delta d\right)-\left(x_{3}\sin\theta +y\cos\theta -y+2\Delta d\right)\right)}{2}\right)\\ \cos\left(\frac{k\left(\left(x_{4}\sin\theta +y\cos\theta -y+2\Delta d\right)+(x_{3}\sin\theta +y\cos\theta -y+2\Delta d)\right)}{2}\right) \end{array}$$

(A13e) $$\sin\left(\frac{ks\sin\theta }{2}\right)\cos\left(\frac{k\left(\left(x_{2}+x_{1}\right)\sin\theta +2y\cos\theta -2y+4\Delta d\right)}{2}\right)-\sin\left(\frac{ks\sin\theta }{2}\right)\cos\left(\frac{k\left(\left(x_{4}+x_{3}\right)\sin\theta +2y\cos\theta -2y+4\Delta d\right)}{2}\right)=0$$

(A14) $$\sin\left(\frac{ks\sin\theta }{2}\right)\left[\cos\left(\frac{k\left(\left(x_{2}+x_{1}\right)\sin\theta +2y\cos\theta -2y+4\Delta d\right)}{2}\right)-\cos\left(\frac{k\left(\left(x_{4}+x_{3}\right)\sin\theta +2y\cos\theta -2y+4\Delta d\right)}{2}\right)\right]=0$$

To obtain the node points that satisfy Equation (A14) for the two intervals defined above, *i.e.*, *x*_2_ – *x*_1_ = *x*_4_ – *x*_3_ = *s*, values need to be assigned to these boundary limits. For example, let *x*_1_, *x*_2_ & *x*_3_, *x*_4_ be the two intervals depicted along the plane of the photodetector in Figure A1 with values defined as *x*_1_ = − *s; x*_2_ = 0; *x*_3_ = − *s* /2; *x*_4_*s*/2.

Substituting these values in Equation (A14) and simplifying yields:

(A14a) $$\sin\left(\frac{ks\sin\theta }{2}\right)\cos\left(\frac{k\left(\left(-s\right)\sin\theta +2y\cos\theta -2y+4\Delta d\right)}{2}\right)-\sin\left(\frac{ks\sin\theta }{2}\right)\cos\left(\frac{k\left(\left(0\right)\sin\theta +2y\cos\theta -2y+4\Delta d\right)}{2}\right)=0$$

(A15) $$\sin\left(\frac{ks\sin\theta }{2}\right)\left[\cos\left(\frac{k\left(\left(-s\right)\sin\theta +2y\cos\theta -2y+4\Delta d\right)}{2}\right)-\cos\left(\frac{k\left(2y\cos\theta -2y+4\Delta d\right)}{2}\right)\right]=0$$

The equality of Equation (A15) is satisfied if

1. $\sin\left(\frac{ks\sin\theta }{2}\right)=0$and/ or
2. $\cos\left(\frac{k\left(\left(-s\right)\sin\theta +2y\cos\theta -2y+4\Delta d\right)}{2}\right)-\cos\left(\frac{k\left(2y\cos\theta -2y+4\Delta d\right)}{2}\right)=0$

Solving:

(a) is satisfied when $\frac{ks\sin\theta }{2}=n_{p}\pi$, where *n_p_* is an integer related to what are termed *primary nodes* (see Figure 3), resulting in:
  (A16) $$\sin\theta_{n_{p}}=\frac{2n_{p}\pi }{ks}\text{or}\theta_{n_{p}}=\frac{n_{p}\lambda }{s}$$
  if small angles are considered, implying *sin θ_np_* = *θ_np_*, where the intersection of the two radiant flux curves at *θ_np_* is called a *primary node* and *θ_np_* is called the *primary node* angle.
(b) is satisfied when
  (A17) $$\cos\left(\frac{k\left(\left(-s\right)\sin\theta +2y\cos\theta -2y+4\Delta d\right)}{2}\right)=\cos\left(\frac{k\left(2y\cos\theta -2y+4\Delta d\right)}{2}\right)$$

There are two solutions that satisfy Equation (A17):

(i). (− *s*) *sin θ* = 0, therefore *sin θ*_0_ = 0 where *θ*_0_ = 0. As only small angles are of concern, *θ* = *nπ* is irrelevant. Note that *θ*_0_ = 0 is co-incident with *primary node n_p_* = 0 from Equation (A16)
(ii). *The* cosines are identical for $n_{s}\pi \pm \frac{\delta }{2}$, where *δ* is a phase shift and *n_s_* is an integer related to *secondary nodes*, *i.e.*,
  (A18) $$\cos\left(n_{s}\pi +\frac{\delta }{2}\right)=\cos\left(n_{s}\pi -\frac{\delta }{2}\right)$$

*n_s_* can be zero if the data is mirrored about *θ* = 0 and *n_s_* = 1 if the data is mirrored about *θ* = *π*.

From Equations (A17) and (A18) there are 2 identities:

(A18a) cosk−ssinθ+2ycosθ−2y+4Δd2=cosnsπ−δ2

(A18b) $$\cos\left(\frac{k\left(2y\cos\theta -2y+4\Delta d\right)}{2}\right)=\cos\left(n_{s}\pi +\frac{\delta }{2}\right)$$

As the left- and right-hand terms refer to the same angle *θ*

(A18c) $$k\left(-s\sin\theta +2y\cos\theta -2y+4\Delta d\right)=2n_{s}\pi -\delta$$

(A18d) $$k\left(2y\cos\theta -2y+4\Delta d\right)=2n_{s}\pi +\delta$$

(A18e) $$k\left(-s\sin\theta +2y\cos\theta -2y+4\Delta d\right)+\delta =2n_{s}\pi$$

(A18f) $$k\left(2y\cos\theta -2y+4\Delta d\right)-\delta =2n_{s}\pi$$

Therefore:

(A18g) k(−ssinθ+2ycosθ−2y+4Δd)+δ=k(2ycosθ−2y+4Δd)−δ

and

(A18h) $$\begin{array}{ll} 2\delta & =k\left(2y\cos\theta -2y+4\Delta d\right)-k\left(-s\sin\theta +2y\cos\theta -2y+4\Delta d\right)\\ & =k2y\cos\theta -k2y+k4\Delta d+ks\sin\theta -k2y\cos\theta +k2y-k4\Delta d\\ & =ks\sin\theta \end{array}$$

The 2*n_s_*π term in Equations (A18e) and (A18f) lies exactly between

(A18i) $$\cos\left(\frac{k\left(\left(-s\right)\sin\theta +2y\cos\theta -2y+4\Delta d\right)}{2}\right)$$

and

(A18j) $$\cos\left(\frac{k\left(2y\cos\theta -2y+4\Delta d\right)}{2}\right)$$

as revealed from Equation (A18h)

Therefore:

(A18k) $$2n_{s}\pi =k\left(2y\cos\theta -2y+4\Delta d\right)-\frac{ks\sin\theta }{2}$$

This eliminates the unknown term Δ and provides the function for *secondary nodes*. Simplifying Equation (A18k) renders:

(A18l) $$2n_{s}\pi =k\left(2y\cos\theta -2y+4\Delta d-\frac{s\sin\theta }{2}\right)$$

(A18m) $$\begin{array}{cc} n_{s}\pi & =k\left(y\cos\theta -y+2\Delta d-\frac{s\sin\theta }{4}\right)\\ & =ky\cos\theta -ky+k2\Delta d-\frac{ks\sin\theta }{4} \end{array}$$

Rearranging and grouping terms yields:

(A18n) $$\frac{ks\sin\theta }{4}=-n_{s}\pi +ky\cos\theta -ky+k2\Delta d$$

(A18o) $$\sin\theta =-\frac{n_{s}\pi }{ks}+\frac{4y\cos\theta }{s}-\frac{4y}{s}+\frac{8\Delta d}{s}$$

(A18p) $$\sin^{2}\theta ={\left(\frac{4n_{s}\pi }{ks}\right)}^{2}+{\left(\frac{4y}{s}\right)}^{2}\cos^{2}\theta +{\left(\frac{4y}{s}\right)}^{2}+{\left(\frac{8\Delta d}{s}\right)}^{2}-\frac{32n_{s}\pi y}{ks^{2}}\cos\theta +\frac{32n_{s}\pi y}{ks^{2}}-\frac{64n_{s}\pi \Delta d}{ks^{2}}-\frac{32y^{2}}{s^{2}}\cos\theta +\frac{64y\Delta d}{s^{2}}\cos\theta -\frac{64y\Delta d}{s^{2}}$$

(A18q) $$\begin{array}{c} \sin^{2}\theta -1={\left(\frac{4n_{s}\pi }{ks}\right)}^{2}+{\left(\frac{4y}{s}\right)}^{2}\cos^{2}\theta +{\left(\frac{4y}{s}\right)}^{2}+{\left(\frac{8\Delta d}{s}\right)}^{2}-\frac{32n_{s}\pi y}{ks^{2}}\cos\theta +\frac{32n_{s}\pi y}{ks^{2}}-\frac{64n_{s}\pi \Delta d}{ks^{2}}-\frac{32y^{2}}{s^{2}}\cos\theta +\frac{64y\Delta d}{s^{2}}\cos\theta -\frac{64y\Delta d}{s^{2}}-1\\ {\left(\frac{4n_{s}\pi }{ks}\right)}^{2}+{\left(\frac{4y}{s}\right)}^{2}\cos^{2}\theta +{\left(\frac{4y}{s}\right)}^{2}+{\left(\frac{8\Delta d}{s}\right)}^{2}-\frac{32n_{s}\pi y}{ks^{2}}\cos\theta +\frac{32n_{s}\pi y}{ks^{2}}-\frac{64n_{s}\pi \Delta d}{ks^{2}}-\frac{32y^{2}}{s^{2}}\cos\theta +\frac{64y\Delta d}{s^{2}}\cos\theta -\frac{64y\Delta d}{s^{2}}-1+1-\sin^{2}\theta =0 \end{array}$$

(A18r) $$\begin{array}{c} {\left(\frac{4n_{s}\pi }{ks}\right)}^{2}+{\left(\frac{4y}{s}\right)}^{2}\cos^{2}\theta +{\left(\frac{4y}{s}\right)}^{2}+{\left(\frac{8\Delta d}{s}\right)}^{2}-\frac{32n_{s}\pi y}{ks^{2}}\cos\theta +\frac{32n_{s}\pi y}{ks^{2}}-\frac{64n_{s}\pi \Delta d}{ks^{2}}-\frac{32y^{2}}{s^{2}}\cos\theta +\frac{64y\Delta d}{s^{2}}\cos\theta -\frac{64y\Delta d}{s^{2}}-1+\cos^{2}\theta =0\\ \left({\left(\frac{4y}{s}\right)}^{2}+1\right)\cos^{2}\theta +\left(\frac{64y\Delta d}{s^{2}}-\frac{32y^{2}}{s^{2}}-\frac{32n_{s}\pi y}{ks^{2}}\right)\cos\theta +\frac{32n_{s}\pi y}{ks^{2}}-\frac{64n_{s}\pi \Delta d}{ks^{2}}+{\left(\frac{4n\pi }{ks}\right)}^{2}+{\left(\frac{4y}{s}\right)}^{2}+{\left(\frac{8\Delta d}{s}\right)}^{2}-\frac{64y\Delta d}{s^{2}}-1=0 \end{array}$$

Let

(A18s) $$A={\left(\frac{4y}{s}\right)}^{2}+1$$

(A18t) $$B=\frac{64y\Delta d}{s^{2}}-\frac{32y^{2}}{s^{2}}-\frac{32n_{s}\pi y}{ks^{2}}$$

(A18u) $$C={\left(\frac{4n_{n_{s}}\pi }{ks}\right)}^{2}+{\left(\frac{4y}{s}\right)}^{2}+{\left(\frac{8\Delta d}{s}\right)}^{2}+\frac{32n_{n_{s}}\pi y}{ks^{2}}-\frac{64n_{n_{s}}\pi \Delta d}{ks^{2}}-\frac{64y\Delta d}{s^{2}}-1$$

then

(A18v) $$A\cos^{2}\theta +B\cos\theta +C=0$$

and

(A18w) $$\cos\theta =\cos_{1,2}\theta_{n_{s}}=\frac{-B\pm \sqrt{B^{2}-4AC}}{2A}$$

By way of demonstrating the presence of *secondary nodes* for the case with integral boundaries defined above as *x*_1_ = − *s*/2; *x*_2_ = 0; *x*_3_ = − *s*/2; *x*_4_ = *s*/2, firstly, assume *Δd* = 0 m.

Equations (A18s), (A18t) and (A18u) now reduce to:

(A18x) $$A={\left(\frac{4y}{s}\right)}^{2}+1$$

(A18y) $$B=-\frac{32y^{2}}{s^{2}}-\frac{32n_{s}\pi y}{ks^{2}}$$

(A18z) $$C={\left(\frac{4n_{s}\pi }{ks}\right)}^{2}+{\left(\frac{4y}{s}\right)}^{2}+\frac{32n_{s}\pi y}{ks^{2}}-1$$

Then, the variables in Equations (A18x)–(A18z) are assigned values, for example those defined in Equation (A18A).

(A18A) $$\begin{array}{l} y=0.02m\\ \lambda =680\cdot 10^{-9}\text{m therefore}k=9239978\\ \text{integral width}s=0.001\text{m} \end{array}$$

Finally, Equation (A18w) is solved for different *n_s_* (positive and negative) to find *θ_ns_* where the radiant flux curves intersect at the *secondary node* points.

*Secondary nodes* for the above defined conditions are returned for

(A18B) $$\cos\theta_{n_{s}1}=\frac{-B+\sqrt{B^{2}-4AC}}{2A}$$

when *n_s_* ≤ 0 and *θ*_ns1_ ≥ 0 and when 0 ≤ *n_s_* ≤ 4 for *θ_ns_*_1_ < 0.

Also

(A18C) $$\cos\theta_{n_{s}2}=\frac{-B-\sqrt{B^{2}-4AC}}{2A}$$

when *n_s_*≤ 4 for *θ_ns_*_2_ < 0.

Consider the solution defined by Equation (A18B) and let n_s_ = ***−*** 1. The *secondary node* point is calculated by this solution, *i.e.*, *θ_−_*_1_ = 0.07409° and confirmed by substituting the above conditions into the integral part of Equation (A8).

Mirror M2 in Figure A1 can tilt left or right of the *y*-axis, therefore *θ_ns_* can be positive or negative, therefore returning *secondary nodes* of both polarities for *θ_ns_* in Equations (A18B) and (A18C).

From the above derivation of the *secondary nodes*, a specific solution was obtained for the defined intervals specified in as *x*_1_ = ***−*** *s; x*_2_ = 0; *x*_3_ = ***−*** *s*/2; *x*_4_ = *s*/2 and the variable values assigned in Equation (A18A). The occurrence of *secondary nodes* is unique and specific to the defined integral boundaries *x*_1_, *x*_2_ & *x*_3_, *x*_4_, the values of *k*, *y* and Δ*d*. Consequently, the cosine expression in Equation (A14) has to be solved accordingly with its own set of boundary conditions, such as Equation (A18A). The wave front angle at *secondary node* angles *θ_ns_* is incidental, unlike at *primary nodes*, which is cyclic and dependent only on *k* and *s* (sine expression in Equation (A14)). The next section derives the magnitude of the radiant flux at the *primary nodes*.

### A3. Determination of the Magnitude of ***Φ_e_*** at Wave Front Angles ***θ_n_***

To determine the magnitude of the radiant flux of the fringe pattern at the *primary nodes* we solve Equations (A12) and (A13) independently for the intervals defined as *x*_1_ = ***−*** *s; x*_2_ = 0; *x*_3_ = ***−*** *s*/2; *x*_4_ = *s*/2. Beginning with Equation (A12):

(A19) $$\begin{array}{ll} \Phi_{e\left(x_{1},x_{2}\right)} & \propto \frac{1}{k\sin\theta }\left[\sin\left(k\left(x_{2}\sin\theta +y\cos\theta -y+2\Delta d\right)\right)+kx_{2}\sin\theta -\sin\left(k\left(x_{1}\sin\theta +y\cos\theta -y+2\Delta d\right)\right)-kx_{1}\sin\theta \right]\\ & \propto \frac{1}{k\sin\theta }\left[\sin\left(k\left(0\sin\theta +y\cos\theta -y+2\Delta d\right)\right)+k0\sin\theta -\sin\left(k\left(-s\sin\theta +y\cos\theta -y+2\Delta d\right)\right)+ks\sin\theta )\right]\\ & \propto \frac{\sin\left(k\left(y\cos\theta -y+2\Delta d\right)\right)-\sin\left(k\left(-s\sin\theta +y\cos\theta -y+2\Delta d\right)\right)+ks\sin\theta }{k\sin\theta } \end{array}$$

For small angles *sin θ* = *θ*, therefore Equation (A19) becomes

(A20) $$\Phi_{e\left(x_{1},x_{2}\right)}\propto \frac{\sin\left(k\left(y\cos\theta -y+2\Delta d\right)\right)-\sin\left(k\left(-s\theta +y\cos\theta -y+2\Delta d\right)\right)+ks\theta }{k\theta }$$

At *θ*_0_, Equation (A20) reduces to 0/0, which is indeterminate. Therefore applying L'Hôpital's rule:

(A21) $$\begin{array}{l} \underset{\theta \to 0}{\lim}\frac{\sin\left(k\left(y\cos\theta -y+2\Delta d\right)\right)-\sin\left(k\left(-s\theta +y\cos\theta -y+2\Delta d\right)\right)+ks\theta }{k\theta }\\ \;=\underset{\theta \to 0}{\lim}\frac{\cos\left(k\left(y\cos\theta -y+2\Delta d\right)\right)\cdot \left(-ky\sin\theta \right)-\cos\left(k\left(-s\theta +y\cos\theta -y+2\Delta d\right)\right)\cdot \left(k\left(-s-y\sin\theta \right)\right)+ks}{k}\\ \;=\underset{\theta \to 0}{\lim}\frac{\cos\left(k\left(y-y+2\Delta d\right)\right)\cdot \left(-ky0\right)-\cos\left(k\left(-s0+y-y+2\Delta d\right)\right)\cdot \left(k\left(-s-y0\right)\right)+ks}{k}\\ \;=\underset{\theta \to 0}{\lim}\frac{-\cos\left(k\left(2\Delta d\right)\right)\cdot \left(k\left(-s\right)\right)+ks}{k}\\ \;=\underset{\theta \to 0}{\lim}\frac{ks\cos\left(k\left(2\Delta d\right)\right)+ks}{k}\\ \;=s\left(\cos\left(k2\Delta d\right)+1\right) \end{array}$$

Repeating the above for Equation (A13)

(A21a) $$\begin{array}{c} \Phi_{e\left(x_{3},x_{4}\right)}\propto \frac{1}{k\sin\theta }\left[\sin\left(k\left(\frac{s}{2}\sin\theta +y\cos\theta -y+2\Delta d\right)\right)+k\frac{s}{2}\sin\theta -\sin\left(k\left(-\frac{s}{2}\sin\theta +y\cos\theta -y+2\Delta d\right)\right)+k\frac{s}{2}\sin\theta )\right]\\ \propto \frac{\sin\left(k\left(\frac{s}{2}\sin\theta +y\cos\theta -y+2\Delta d\right)\right)+ks\sin\theta -\sin\left(k\left(-\frac{s}{2}\sin\theta +y\cos\theta -y+2\Delta d\right)\right)}{k\sin\theta } \end{array}$$

For small angles *sin θ* = *θ*, therefore Equation (A21a) becomes

(A21b) $$\Phi_{e\left(x_{3},x_{4}\right)}\propto \frac{\sin\left(k\left(\frac{s}{2}\theta +y\cos\theta -y+2\Delta d\right)\right)+ks\theta -\sin\left(k\left(-\frac{s}{2}\theta +y\cos\theta -y+2\Delta d\right)\right)}{k\theta }$$

At *θ*_0_, Equation (A20) reduces to $\frac{0}{0}$, which again is indeterminate. Applying L'Hôpital's rule

(A21c) $$\begin{array}{l} \;\underset{\theta \to 0}{\text{lim}}\frac{\sin\left(k\left(\frac{s}{2}\theta +y\cos\theta -y+2\Delta d\right)\right)+ks\theta -\sin\left(k\left(-\frac{s}{2}\theta +y\cos\theta -y+2\Delta d\right)\right)}{k\theta }\\ =\underset{\theta \to 0}{\text{lim}}\frac{\cos\left(k\left(\frac{s}{2}\theta +y\cos\theta -y+2\Delta d\right)\right)\cdot k\left(\frac{s}{2}-y\sin\theta \right)+ks-\cos\left(k\left(-\frac{s}{2}\theta +y\cos\theta -y+2\Delta d\right)\right)\cdot k\left(-\frac{s}{2}-y\sin\theta \right)}{k}\\ =\underset{\theta \to 0}{\text{lim}}\frac{\cos\left(k2\Delta d\right)\cdot k\left(\frac{s}{2}\right)+kx-\cos\left(k2\Delta d\right)\cdot k\left(-\frac{s}{2}\right)}{k}\\ =\underset{\theta \to 0}{\text{lim}}\frac{ks\cos\left(k2\Delta d\right)+ks}{k}=s(cos(k2\Delta d)+1) \end{array}$$

Equations (A21) and (A21c) show that as *θ* → 0 the radiant flux becomes equal for the integral boundaries defined as *x*_1_ = ***−*** *s; x*_2_ = 0; *x*_3_ = ***−*** *s*/2; *x*_4_ = *s*/2. Substituting the result of Equation (A21) or Equation (A21c) into Equation (A8) gives Equation (A22), which shows the magnitude of the radiant flux as *θ*→0 is dependent on *k* and Δ*d* and independent of *y*.

(A22) $$\Phi_{e\left(\theta \to 0\right)}=2\left(\frac{n_{RI}\epsilon_{0}c}{2}\right){E_{0}}^{2}z\cdot s(cos(k2\Delta d)+1)$$

Note Equations (A10) and (A22) are equal, resulting in maximum radiant flux for translations where 2Δ*d* = *nλ*.

(A23) $$\Phi_{e\left(\theta \to 0,2\Delta d=n\lambda \right)}=2\left(\frac{n_{RI}\epsilon_{0}c}{2}\right){E_{0}}^{2}z\cdot 2s$$

To work out the value of radiant flux for all other values of *θ_np_*, *i.e.*, *θ*_1, 2, 3,.._, substitute inline from Equation (A16) into Equation (A20) and Equation (A21b) independently. Beginning with Equation (A20) and using the identity in Equation (A13b):

(A23a) $$\begin{array}{l} \Phi_{e\left(x_{1},x_{2}\right)}\\ \propto \frac{\sin\left(k\left(y\cos\left(\frac{n_{p}\lambda }{s}\right)-y+2\Delta d\right)\right)-\sin\left(k\left(-s\frac{n_{p}\lambda }{s}+y\cos\left(\frac{n_{p}\lambda }{s}\right)-y+2\Delta d\right)\right)+ks\frac{n_{p}\lambda }{s}}{k\frac{n\lambda }{s}}\\ \propto \frac{\sin\left(k\left(y\cos\left(\frac{n_{p}\lambda }{s}\right)-y+2\Delta d\right)\right)-\sin\left(k\left(-n_{p}\lambda +y\cos\left(\frac{n_{p}\lambda }{s}\right)-y+2\Delta d\right)\right)+kn\lambda }{k\frac{n\lambda }{s}}\\ \propto \frac{s}{kn_{p}\lambda }\left[2\sin\left(\frac{k\left(y\cos\left(\frac{n_{p}\lambda }{s}\right)-y+2\Delta d\right)-k\left(-n_{p}\lambda +y\cos\left(\frac{n_{p}\lambda }{s}\right)-y+2\Delta d\right)}{2}\right)\cdot \cos\left(\frac{k\left(y\cos\left(\frac{n_{p}\lambda }{s}\right)-y+2\Delta d\right)+k\left(-n_{p}\lambda +y\cos\left(\frac{n_{p}\lambda }{s}\right)-y+2\Delta d\right)}{2}\right)+kn_{p}\lambda \right]\\ \propto \frac{s}{kn_{p}\lambda }\left[2\sin\left(\frac{kn_{p}\lambda }{2}\right)\cdot \cos\left(k\left(y\cos\left(\frac{n_{p}\lambda }{s}\right)-y+2\Delta d-\frac{n_{p}\lambda }{2}\right)\right)+kn_{p}\lambda \right]\\ \propto \frac{s}{kn_{p}\lambda }\left[2\sin\left(n_{p}\pi \right)\cdot \cos\left(k(ycos\left(\frac{n_{p}\lambda }{s}\right)-y+2\Delta d-\frac{n_{p}\lambda }{s})\right)+kn_{p}\lambda \right]\\ \propto s \end{array}$$

Repeating the above for Equation (A21b)

(A24) $$\begin{array}{l} \Phi_{e\left(x_{3},x_{4}\right)}\\ \propto \frac{\sin\left(k\left(\frac{s}{2}\frac{n_{p}\lambda }{s}+y\cos\left(\frac{n_{p}\lambda }{s}\right)-y+2\Delta d\right)\right)+ks\frac{n_{p}\lambda }{s}-\sin\left(k\left(-\frac{s}{2}\frac{n_{p}\lambda }{s}+y\cos\left(\frac{n_{p}\lambda }{s}\right)-y+2\Delta d\right)\right)}{k\frac{n_{p}\lambda }{s}}\\ \;\propto \frac{\sin\left(k\left(\frac{n_{p}\lambda }{2}+y\cos\left(\frac{n_{p}\lambda }{s}\right)-y+2\Delta d\right)\right)+kn_{p}\lambda -\sin\left(k\left(-\frac{n_{p}\lambda }{2}+y\cos\left(\frac{n_{p}\lambda }{s}\right)-y+2\Delta d\right)\right)}{\frac{kn\lambda }{s}}\\ \;\propto \frac{s}{kn_{p}\lambda }\left[2\sin\left(\frac{k\left(\frac{n_{p}\lambda }{2}+y\cos\left(\frac{n_{p}\lambda }{s}\right)-y+2\Delta d\right)-k\left(-\frac{n_{p}\lambda }{2}+y\cos\left(\frac{n_{p}\lambda }{s}\right)-y+2\Delta d\right)}{2}\right)\cdot \cos\left(\frac{k\left(\frac{n_{p}\lambda }{2}+y\cos\left(\frac{n_{p}\lambda }{s}\right)-y+2\Delta d\right)+k\left(-\frac{n_{p}\lambda }{2}+y\cos\left(\frac{n_{p}\lambda }{s}\right)-y+2\Delta d\right)}{2}\right)+kn_{p}\lambda \right]\\ \;\propto \frac{s}{kn_{p}\lambda }\left[2\sin\left(\frac{kn_{p}\lambda }{2}\right)\cdot \cos\left(y\cos\left(\frac{n_{p}\lambda }{s}\right)-y+2\Delta d\right)+kn_{p}\lambda \right]\\ \;\propto \frac{s}{kn_{p}\lambda }\left[2\sin\left(n_{p}\pi \right)\cdot \cos\left(y\cos\left(\frac{n_{p}\lambda }{s}\right)-y+2\Delta d\right)+kn_{p}\lambda \right]\\ \;\propto s \end{array}$$

Equations (A23a) and (A24) give the same result for the integral of the active areas at *θ_np_* = *θ*_1,2,3,.._ and therefore the radiant flux at these wave front angles is given by Equation (A25).

(A25) $$\Phi_{e\left(\theta_{1,2,3,\ldots }\right)}=2\left(\frac{n_{RI}\epsilon_{0}c}{2}\right){E_{0}}^{2}z\cdot s$$

Equation (A25) also shows that the radiant flux at *θ_np_* = *θ*_1,2,3,.._ is half maximum (cf. Equation (A23)), and in contrast to the radiant flux at *θ_np_* = *θ*_0_ given in Equation (A22), *Φ_e_*(*θ*_1,2,3,.._) is independent of *k* and Δ*d*. The reason for this is the width *s* of the active area is an integer multiple of the fringe line spacing at *primary node* angles *θ_np_* = *θ*_1,2,3,…_

When there is an exact multiple of fringe lines within the active area, the radiant flux across the active area is the mean of the maximum constructive and destructive interferences, *i.e.*, 50 %. This means that if the active area is moved in either direction along the *x*-axis, the radiant flux remains static at 0.5 normalised magnitude. If mirror M2 translates, there will be no change in the radiant flux despite the fringe pattern moving back and forth across the active area.

### A4. Effect of Distance x and y of Photodetector from Origin with Varying θ

To determine the effect of distance *y* of the photodetector from the origin with varying *θ*, the position and slope of the fringe lines needs to be determined. This is done by finding the instances of maximum value of irradiance in Equation (A7), *i.e.*, when

(A26) cos(k(y−2Δd−ycosθ−xsinθ))=1

Equation (A26) is true when

(A27) $$k\left(y-2\Delta d-y\cos\theta -x\sin\theta \right)=2n_{f}\pi$$

where *n_f_* is the *n^th^* fringe line. Solving for *y* gives:

(A27a) $$y\left(1-\cos\theta \right)=x\sin\theta +n_{f}\lambda +2\Delta d$$

(A28) $$y=\frac{x\sin\theta }{\left(1-\cos\theta \right)}+\frac{n_{f}\lambda +2\Delta d}{\left(1-\cos\theta \right)}$$

Equation (A28) is a linear equation and defines the profile of the fringe pattern in the *x-y* plane as illustrated in Figure A1 where $\frac{\sin\theta }{\left(1-\cos\theta \right)}$ is the slope of the fringe lines and $\frac{n_{f}\lambda +2\Delta d}{\left(1-\cos\theta \right)}$ is the *y* intercept. As only small angles are being considered it becomes indeterminate at *θ* = 0, which stands to reason as the fringe pattern is uniform across the active area as well as the *x-y* plane and no fringe lines are present.

By solving Equation (A28) for *n_f_*, the number of fringes lines passing over a given point (*x,y*) can be calculated for *θ* increasing or decreasing from zero to a given wave front angle.

(A28a) $$n_{f}\lambda =y\left(1-\cos\theta \right)-x\sin\theta -2\Delta d$$

(A29) $$n_{f}=\frac{y\left(1-\cos\theta \right)}{\lambda }-\frac{x\sin\theta }{\lambda }-\frac{2\Delta d}{\lambda }$$

The *y* term in the above equation is positive for *θ* ≠ 0 and is symmetrical in shape as a function of *θ*.

For small angles *sin θ* = *θ*, therefore the coefficient of *x* is a linear function of angle *θ*.

The polarity of *n_f_* is therefore dependent on the magnitude and sign of *x, θ* and Δ*d*.

When *θ*= 0, Equation (A29) reduces to

(A29a) $$-2\Delta d=n_{f}\lambda$$

*n_f_* is the number of fringes counted at a point (*x, y*) as *θ* is varied. Assume Δ*d* = 0, there is a special case in Equation (A29) when:

(A30) $$\frac{y\left(1-\cos\theta \right)}{\lambda }=\frac{x\sin\theta }{\lambda }$$

and the fringe count *n_f_* = 0 despite *θ* > 0. In this case, fringe lines would have moved over point (*x, y*) in one direction as *θ* is increased and then back again as *θ* is increased further to the angle *θ* that satisfies Equation (A30).

As discussed with Equation (A28), the slope of the fringe lines is given by:

(A31) $$\frac{y}{x}=\frac{\sin\theta }{\left(1-\cos\theta \right)}=\cot\left(\frac{\theta }{2}\right)$$

where $\frac{\theta }{2}$ is the angle the normal of mirror M2 relative to the *y*-axis (Figure 3). Therefore, there is a set of points (*x, y*) coincident with the mirror normal that renders *n_f_* = 0.

#### Deriving the speed of fringe movement

If *Δd* is considered static (Δ*d* = *0*), Equation (A29) reduces to

(A32) $$n_{f}=\frac{y\left(1-\cos\theta \right)}{\lambda }-\frac{x\sin\theta }{\lambda }$$

Taking the derivative of the *x*-term delivers the speed of concertinaing of the fringe lines at the point (*x, y*).

(A33) $$\frac{\text{d}n_{f}}{\text{d}\sin\theta }=-\frac{x}{\lambda }$$

The speed of sideways deflection of the fringe lines at point (*x, y*) results from calculating the derivative of the *y*-term

(A34) $$\frac{\text{d}n_{f}}{\text{d}\sin\theta }=\frac{y}{\lambda }\frac{\sin\theta }{\sqrt{1-\sin^{2}\theta }}=\frac{y}{\lambda }\tan\theta$$

The overall fringe movement speed is therefore

(A35) $$\frac{\text{d}n_{f}}{\text{d}\sin\theta }=-\frac{x}{\lambda }+\frac{y}{\lambda }\tan\theta$$

From Equation (A35), the direction of fringe movement reverses if

(A36) $$-\frac{x}{\lambda }+\frac{y}{\lambda }\tan\theta =0$$

And therefore the angle of movement reversal *θ_rev_* is

(A37) $$\theta_{\text{rev}}=\tan^{-1}\frac{x}{y}=\sin^{-1}\frac{x}{\sqrt{x^{2}+y^{2}}}$$

That is, when the perpendicular of the wave front from the origin points towards +*x*. The mirror tilt angle is therefore *θ_rev_*/2. If *y* → 0, *θ_rev_* → *π*/2, *i.e.*, the smaller *y*, the larger is *θ_rev_*.

From Equation (A29), the fringe count returns to zero if the numerical value of *x*- and *y*-terms cancel each other out.

(A38) y−ycosθ−xsinθ=0

(A38a) $$1-\frac{x}{y}\sin\theta =\cos\theta$$

(A38b) $$1-\frac{x}{y}\sin\theta =\sqrt{1-\sin^{2}\theta }$$

(A38c) $$1+\frac{x^{2}}{y^{2}}\sin^{2}\theta -2\frac{x}{y}\sin\theta =1-\sin^{2}\theta$$

(A38d) $$\frac{x^{2}}{y^{2}}\sin^{2}\theta -2\frac{x}{y}\sin\theta =-\sin^{2}\theta$$

(A38e) $$\frac{x^{2}}{y^{2}}\sin\theta -2\frac{x}{y}=-\sin\theta$$

(A38f) $$\frac{x^{2}}{y^{2}}\sin\theta +\sin\theta =2\frac{x}{y}$$

(A38g) $$\sin\theta \left(\frac{x^{2}}{y^{2}}+1\right)=2\frac{x}{y}$$

(A39) $$\sin\theta =\frac{2\frac{x}{y}}{\left(\frac{x^{2}}{y^{2}}+1\right)}=\frac{2\frac{x}{y}}{\left(\frac{x^{2}+y^{2}}{y^{2}}\right)}=\frac{2xy^{2}}{y\left(x^{2}+y^{2}\right)}=\frac{2xy}{\left(x^{2}+y^{2}\right)}$$

This occurs at the angle *θ_n_*_= 0_

(A40) $$\theta_{n=0}=\sin^{-1}\frac{2xy}{\left(x^{2}+y^{2}\right)}$$

If *x* = *y*, then $\theta_{n=0}=\frac{\pi }{2}$, if *x* > *y*, then $\theta_{n=0}>\frac{\pi }{2}$ and conversely, if *y* > *x*, then $\theta_{n=0}<\frac{\pi }{2}$.

The relationship between *θ_n_*_= 0_ and *θ_rev_*, results in the identity *θ_n_*_= 0_ ≡ 2*θ_rev_*.

From Equations (A37) and (A40)

(A41) $$\theta_{\text{rev}}=\frac{1}{2}\sin^{-1}\frac{2xy}{\left(x^{2}+y^{2}\right)}=\sin^{-1}\frac{x}{\sqrt{x^{2}+y^{2}}}$$

Resulting in

(A42) $$\sin2\theta_{\text{rev}}=2\sin\theta_{\text{rev}}\cos\theta_{\text{rev}}=\frac{2xy}{\left(x^{2}+y^{2}\right)}$$

And proving the identity of *θ_n_*_= 0_ ≡ 2*θ_rev_* being correct.

#### Determination of the damping function of the flux curve

From Figure 4 it is evident that the normalised flux curve oscillates about a level of 0.5 flux and decreases its wave amplitude about this level with increasing wave front angle *θ*. As this behaviour constitutes a damped function, the flux decay function is derived subsequently.

For a centred photodetector of width *s* and positioned at *x* = 0, with integral boundaries ${\text{x}}_{2}=\frac{+s}{2}$ & ${\text{x}}_{1}=\frac{-s}{2}$, *y* = 0 and Δ*d* = 0, the radiant flux *Φ_e_*_(_*_x_*_1_,*_x_*_2)_ in Equation (A8) reduces to

(A43) $$\begin{array}{ll} \Phi_{e\left(x_{1},x_{2}\right)} & \propto \frac{1}{k\sin\theta }\left[\sin\left(\frac{ks\sin\theta }{2}\right)+\frac{ks\sin\theta }{2}-\sin\left(\frac{-ks\sin\theta }{2}\right)-\frac{-ks\sin\theta }{2}\right]\\ & \propto \frac{1}{k\sin\theta }\left[2\sin\left(\frac{ks\sin\theta }{2}\right)+ks\sin\theta \right]\\ & \propto \frac{2\sin\left(\frac{ks\sin\theta }{2}\right)}{k\sin\theta }+s \end{array}$$

In Equation (A43), the maximal flux corresponds to 2*s* (*cf.* Equations (A11) and (A23): flux = constant * *z* * 2*s; z* is considered unity as the *z*-direction is perpendicular to fringe lines).

Normalising the flux to 2*s* delivers the normalised flux *Φ_n_*

(A44) $$\begin{array}{cc} \Phi_{n} & \propto \frac{2\sin\left(\frac{ks\sin\theta }{2}\right)}{2sk\sin\theta }+\frac{s}{2s}\\ & \propto \frac{\sin\left(\frac{ks\sin\theta }{2}\right)}{sk\sin\theta }+\frac{1}{2} \end{array}$$

As the normalised flux ranges from 0 to +1, it is converted to a range from −1 to +1 in order to compare it to a standard sine function

(A45) $$\begin{array}{ll} \Phi_{n} & \propto 2\left[\frac{\sin\left(\frac{ks\sin\theta }{2}\right)}{sk\sin\theta }+\frac{1}{2}\right]-1\\ & \propto 2\frac{\sin\left(\frac{ks\sin\theta }{2}\right)}{sk\sin\theta }\\ & \propto \frac{\sin\left(\frac{ks\sin\theta }{2}\right)}{\frac{ks\sin\theta }{2}}\\ & \propto \text{sinc}\left(\frac{ks\sin\theta }{2}\right) \end{array}$$

For small *θ*, Equation (A45) becomes

(A45a) $$\Phi_{n}\propto \text{sinc}\left(\frac{ks\theta }{2}\right)$$

This equation defines a damped sine function. The equivalent undamped sine function has the form of

(A45b) A=sin(2πfθ)

Where *A* is the amplitude and *f* is the reciprocal value of 2nd node angle ( $=2\frac{\lambda }{s}$), *i.e.*, the wave length of the flux function. Substituting this term into Equation (A45b) yields

(A45c) $$A=\sin\left(2\pi \frac{s}{2\lambda }\theta \right)=\sin\left(\frac{\pi s\theta }{\lambda }\right)$$

The decay function *F_D_* is obtained when normalising the damped sine function to its undamped counterpart

(A45d) $$F_{D}=\frac{2\frac{\sin\left(\frac{ks\sin\theta }{2}\right)}{sk\sin\theta }}{\sin\left(\frac{\pi s\theta }{\lambda }\right)}=\frac{2\sin\left(\frac{ks\sin\theta }{2}\right)}{sk\sin\theta \sin\left(\frac{\pi s\theta }{\lambda }\right)}$$

After simplifying and considering that *θ* is small

(A45e) $$F_{D}=\frac{2}{sk\theta }\cdot \frac{\sin\left(\frac{ks\theta }{2}\right)}{\sin\left(\frac{\pi s\theta }{\lambda }\right)}$$

Considering that $k=\frac{2\pi }{\lambda }$

(A46) $$F_{D}=\frac{2}{sk\theta }\frac{\sin\left(\frac{ks\theta }{2}\right)}{\sin\left(\frac{ks\theta }{2}\right)}=\frac{2}{sk}\cdot \frac{1}{\theta }=\frac{\lambda }{\pi s}\cdot \frac{1}{\theta }$$

Equation (A46) is the constitutive equation of the flux decay function at *x* = 0 and *y* = 0. *F_D_* is a reciprocal function of *θ*, with a decay constant *C_D_* of $\frac{2}{sk}$ or $\frac{\lambda }{\pi s}$.
